# Genome-wide analysis of genetic predisposition to Alzheimer’s disease and related sex disparities

**DOI:** 10.1186/s13195-018-0458-8

**Published:** 2019-01-12

**Authors:** Alireza Nazarian, Anatoliy I. Yashin, Alexander M. Kulminski

**Affiliations:** Biodemography of Aging Research Unit, Social Science Research Institute, Duke University, Erwin Mill Building, 2024 W. Main St., Durham, NC 27705 USA

**Keywords:** Alzheimer’s disease, Sex disparities, Genome-wide association study, Meta-analysis, Gene-based analysis

## Abstract

**Background:**

Alzheimer’s disease (AD) is the most common cause of dementia in the elderly and the sixth leading cause of death in the United States. AD is mainly considered a complex disorder with polygenic inheritance. Despite discovering many susceptibility loci, a major proportion of AD genetic variance remains to be explained.

**Methods:**

We investigated the genetic architecture of AD in four publicly available independent datasets through genome-wide association, transcriptome-wide association, and gene-based and pathway-based analyses. To explore differences in the genetic basis of AD between males and females, analyses were performed on three samples in each dataset: males and females combined, only males, or only females.

**Results:**

Our genome-wide association analyses corroborated the associations of several previously detected AD loci and revealed novel significant associations of 35 single-nucleotide polymorphisms (SNPs) outside the chromosome 19q13 region at the suggestive significance level of *p* < 5E–06. These SNPs were mapped to 21 genes in 19 chromosomal regions. Of these, 17 genes were not associated with AD at genome-wide or suggestive levels of associations by previous genome-wide association studies. Also, the chromosomal regions corresponding to 8 genes did not contain any previously detected AD-associated SNPs with *p* < 5E–06. Our transcriptome-wide association and gene-based analyses revealed that 26 genes located in 20 chromosomal regions outside chromosome 19q13 had evidence of potential associations with AD at a false discovery rate of 0.05. Of these, 13 genes/regions did not contain any previously AD-associated SNPs at genome-wide or suggestive levels of associations. Most of the newly detected AD-associated SNPs and genes were sex specific, indicating sex disparities in the genetic basis of AD. Also, 7 of 26 pathways that showed evidence of associations with AD in our pathway-bases analyses were significant only in females.

**Conclusions:**

Our findings, particularly the newly discovered sex-specific genetic contributors, provide novel insight into the genetic architecture of AD and can advance our understanding of its pathogenesis.

**Electronic supplementary material:**

The online version of this article (10.1186/s13195-018-0458-8) contains supplementary material, which is available to authorized users.

## Background

Alzheimer’s disease (AD) is a slowly progressive neurodegenerative disorder that usually manifests with insidious deterioration of cognitive functions such as memory, language, judgment, and reasoning. Visuospatial deficits and neuropsychiatric symptoms like anxiety, irritability, depression, delusion, and personality changes may occur in the course of the disease, and these are eventually followed by impairment of most daily activities [1, 2]. The median survival is 3.3–11.7 years after disease manifestation [3]. Except for some uncommon autosomal dominant forms, AD is mainly a complex disorder with a polygenic nature [2, 4] that predominantly affects elderly individuals, also known as late-onset AD. It is the most common cause of dementia in the elderly worldwide [5] and is the sixth leading cause of death in the United States [6]. Age is the main risk factor for AD. The annual incidence increases from 1% at age 65 years to 6–8% after 85 years [7], and its prevalence increases from 11% to 32% [5]. In addition, AD is more prevalent in females than males [7–10], with their lifetime risk of developing the disease being almost twice that of males [7]. This might be to some extent justified by different life expectancies of males and females. However, Genin et al. [11] suggested that the age-adjusted penetrance of *Apolipoprotein E* (*APOE*) was sex dependent as well. For instance, they found that the lifetime risks for homozygote *APOE-ε4* carriers were 51% and 60% in males and females older than 85 years, respectively. The corresponding risks for heterozygote *APOE-ε3ε4* carriers were 23% and 30%, respectively [11]. AD is also more severe in females than males [9]. Henderson and Buckwalter [12] reported that female AD patients had greater impairment of naming task, verbal fluency, and delayed recall compared to male patients. In another study, Barnes et al. [13] suggested that females were more likely to develop clinical AD compared to males in response to pathology changes (e.g., amyloid beta (Aβ) and neurofibrillary tangles) in the brain. They found that each additional unit of pathology in the brain would increase the odds of overt AD by 20-fold and 3-fold in females and males, respectively [13]. The underlying mechanisms of sex disparity in AD are not fully clear [9, 14]. This may raise the possibility that such sex disparities might be in part due to potential differences in the genetic bases of AD between males and females. Investigating such differences is important, particularly for tailoring more effective medical interventions [14, 15].

Give the considerable physical, emotional, and economic burdens imposed by AD on patients, their families, and societies, exploring the genetic and nongenetic mechanisms underlying its pathogenesis has become a public health priority. With increased life expectancy, the prevalence and global economic costs of AD are forecast to increase considerably by 2050 [5]. Many studies have investigated the genetic basis of AD. *APOE* was the first gene linked to late-onset AD [16], and, in particular, the dosage of its *ε4* allele was implicated in increasing the risks of disease and earlier onset [17]. More susceptibility loci were detected with the advent of genome-wide association (GWA) methodology, although not all of them were consistently replicated in independent datasets. In addition to *APOE*, which was almost universally replicated, *BIN1*, *CLU*, *CR1*, *CD2AP*, *CD33*, *MS4A4E*, *MS4A6A, EPHA1*, and *PICALM* genes have been associated with the polygenic form of AD in different studies [18, 19]. The narrow-sense heritability (*h*^2^) of AD (i.e., the proportion of its phenotypic variance explained by additive genetic variance) has been estimated to be 58–79% by twin studies [20]. Furthermore, Ridge et al. [19], using a linear mixed models (LMMs) framework, found that 53% of phenotypic variance of AD can be explained by ~ 8 million single-nucleotide polymorphisms (SNPs). They also noticed that SNPs inside known AD-associated genes or within their 50 kb upstream/downstream regions can only explain ~ 31% of AD phenotypic variance (~ 59% of genetic variance) [19], leaving a sizable portion of its *h*^2^ to be explained.

In this study, we investigated the genetic architecture of polygenic AD through genome-wide association (GWA), transcriptome-wide association (TWA), gene-based, and pathway-based analyses in four independent datasets (two with family designs and two with population designs) using genetic information for approximately 2 million genotyped and imputed SNPs. Since exploring the genetic sex disparity of AD was of particular interest, in addition to analyzing the entire sample of males and females in each dataset, two alternative plans were also considered in which either only males or only females were included in analyses.

## Methods

### Study participants

Four independent datasets were used to fulfill the aims of this study: Late-Onset Alzheimer’s Disease Family Study from the National Institute on Aging (NIA-LOADFS) [21]; Framingham SNP Health Association Resource (SHARe) project from Framingham Heart Study (FHS) [22–24]; SNP Typing for Association with Multiple Phenotypes from Existing Epidemiologic Data (STAMPEED) project from Cardiovascular Health Study (CHS) [25]; and University of Michigan Health and Retirement Study (HRS) [26]. All four datasets were approved by the institutional review boards (IRBs) and had gathered data after obtaining written informed consent from participants or their legal guardians/proxies. Details about the designs of the NIA-LOADFS, FHS, CHS, and HRS studies can be found in the original publications. Briefly, the NIA-LOADFS is a family-based study primarily initiated to investigate late-onset AD risk factors. It recruited families with multiple affected members if the age at AD onset or diagnosis of proband was above 60 years. Controls were selected from unaffected individuals with a minimum age of 50 years who had no history of major neurological/psychiatric disorders or life-threatening conditions. Of 9468 participants with phenotype data, 5220 subjects (2319 affected with AD), predominantly Caucasians, were genotyped using Illumina’s Human 610*-*Quad array. The FHS is an ongoing longitudinal study with a family-based design that provides phenotype and genotype information on individuals from three-generational families with Caucasian ancestry. The main objective of the study was to investigate cardiovascular disorder risk factors. It was first initiated by recruiting 5209 participants (i.e., original cohort) between ages 30 and 62 years with no history of cardiac disease or stroke. Later, the cohort was expanded by adding the offspring of the original cohort and their spouses (5124 subjects as the offspring cohort) and their grandchildren (4095 subjects as the third generation). Of these, 9274 individuals (1529, 3852, and 3893 individuals from the three aforementioned generations, respectively) were genotyped using the Affymetrix Human Mapping 500 K array in the SHARe project. The CHS is a population-based longitudinal study with the main objective of investigating risk factors contributing to heart diseases. It was initiated by recruiting an original cohort of 5221 mainly Caucasian participants who were older than 65 years and had not been institutionalized. Later, a new cohort of 687 participants, predominantly African-Americans, was added to the study. Of these, 3989 and 803 individuals were genotyped by Illumina’s Human CNV370-Duo and Human Omni1-Quad arrays, respectively, in the STAMPEED project. The HRS is a population-based longitudinal study launched to provide age-related health and economic information on more than 20,000 individuals older than 50 years. The HRS makes use of administrative records such as Social Security and Medicare claims to gather information of interest about participants. The study was expanded in 2006 to include a biomarker and genetic component in which 12,595 individuals, predominantly Caucasian, were genotyped by Illumina’s Human Omni2.5-Quad array.

Our study focused on people of Caucasian ancestry from the four aforementioned studies to increase the sample size and power of the analyses. The LOADFS and FHS datasets directly identify cases with Alzheimer’s disease and unaffected controls. For the CHS and HRS datasets, the International Classification of Disease codes, ninth revision (ICD-9) were used to define cases and controls. Finally, to make the four datasets comparable in terms of participants age, we only included the original and offspring cohorts from the FHS dataset. Demographic information about the cohorts included in our study is presented in Table 1. Also, Additional file 1: Table S1 lists the numbers of cases and controls in these cohorts.

**Table 1:** Demographic information about the four cohorts under consideration

Cohort	Total	Female%	Case%	Age_Case_ (SD)	Age_Control_ (SD)
LOADFS	3716	62.43	49.78	85.93 (8.39)	71.19 (11.53)
FHS	4409	54.77	9.37	79.85 (8.49)	62.77 (11.65)
CHS	3197	60.28	6.16	74.76 (5.36)	73.01 (5.57)
HRS	6158	57.31	4.56	80.44 (6.71)	73.69 (7.85)

*Female%* percentage of females in cohort, *Case%* percentage of patients with Alzheimer’s disease in cohort *Age* average age, *SD* standard deviation, *LOADFS* Late-Onset Alzheimer’s Disease Family Study from the National Institute on Aging, *FHS* Framingham Heart Study, *CHS* Cardiovascular Health Study, *HRS* University of Michigan Health and Retirement Study

### Imputation of genotype data

Since the four datasets of interest were genotyped using different platforms, imputation was conducted to generate a common set of 2,928,658 SNPs. Only autosomal SNPs were subject to imputation. Genome coordinates of SNPs in our data (NCBI build 38/UCSC hg38) were lifted over to NCBI build 37/UCSC hg19 using LiftOver software [27]. After removing duplicate SNPs, preimputation quality control (QC) was performed using PLINK software [28] to remove low-quality SNPs/subjects by setting the following QC criteria: minor allele frequency < 0.01, SNPs and subject call rates < 95%, and Hardy–Weinberg *p* < 1E–06. For the LOADFS and FHS cohorts that have family-based designs, a Mendel error rate of 2% was set to remove SNPs and subjects/families with high Mendelian errors. The SHAPEIT2 (i.e., Segmented Haplotype Estimation and Imputation Tool) package [29] was used to ensure that alleles were aligned to the same DNA strand in our and the reference data. Haplotype phasing was then conducted using SHAPEIT2 to estimate the haplotypes for subjects in each dataset. Finally, genotypes were imputed by Minimac3 software [30] over prephased haplotypes. SHAPEIT2 and Minimac3 were run using default values for input arguments and European population (EUR) haplotypes from 1000 Genomes Phase 3 data (release October 2014) as the reference panel.

### Postimputation QC

Directly genotyped SNPs along with the imputed SNPs, for which the squared correlation (*r*^2^) between imputed and expected true genotypes was > 0.7, were selected for preanalysis QC. This step was performed based on the same criteria explained earlier for preimputation QC. Additional file 1: Table S2 contains information on the numbers of genotyped and imputed SNPs that remained in each of the four datasets of interest after QC.

### Population structure

The top 20 principal components (PCs) of genotype data were obtained through principal component analysis (PCA) to be included in downstream genetic analyses to address potential population stratification. In each dataset, PCA was performed over a subset of unrelated individuals and a subset of SNPs that were not in high linkage disequilibrium (LD) measured by *r*^2^ [31]. KING (i.e., Kinship-based Inference for Genome-wide association studies) software [32] was used to obtain the subset of unrelated subjects by keeping one subject per family or relative cluster whose identity-by-descent (IBD) was > 0.0884 (i.e., closer than third-degree relatives). The genotyped autosomal SNPs on each chromosome were then pruned by PLINK software [28] in an unrelated set of subjects such that no SNP pairs with *r*^2^ > 0.2 were kept within any 100-SNP windows. PCA was then conducted over the selected low-LD SNPs with the GENESIS R package [33, 34]. Additional file 1: Table S3 contains genomic inflation factors (*λ* values) resulting from logistic regression models for the four datasets under consideration. The *λ* values were less than 1.1 in all cases, indicating a subtle impact of population structure on our analyses [35, 36].

### Genetic analysis

#### GWA analysis

The associations between SNPs and AD were investigated by fitting logistic regression models. The genetic analyses of each dataset were performed under three alternative plans analyzing the entire sample, only males, and only females. The top five PCs and subject’s birth cohort (i.e., birth year) were included in the models as fixed-effects covariates. In addition, sex was considered a fixed-effect covariate under plan 1. Only additive genetic effects were modeled; dominance effects were ignored. The birth cohort is a proxy for the age and environmental exposures which are characteristic for a cohort. Thus, this adjustment controls for age and overtime trends in the incidence of AD. The logistic models were fitted using PLINK software (v1.07) [28]. It was previously suggested that for samples with a family-based design, ignoring family relationships would not generate considerable bias in effect sizes of SNPs but may increase type I error rates whose magnitude depends on pedigree complexity (e.g., nuclear family vs extended family) and trait heritability. For instance, the inflation of type I error rates has been suggested to be trivial in datasets with simple pedigrees. On the other hand, type I error rates may increase by a factor of 2–3 when family structure is ignored in a dataset with an extended family pedigree and trait heritability values of 0.6–0.9. Therefore, a two-step screening–validating approach could be used with such datasets to prevent inflation of type I error rates and decrease the computational burden of analysis [37]. For the LOADFS and FHS cohorts, we adopted a two-step approach in which the SNPs with *p* < 0.05 in the logistic models explained earlier were subjected to fitting generalized linear mixed models (GLMMs) by including all aforementioned fixed-effects covariates along with family IDs as a random-effects covariate. GLMMs were fitted using the lme4 R package [33, 38].

All GWA analyses were conducted in a discovery–replication manner. Each of the LOADFS, FHS, CHS, and HRS datasets was considered a discovery set to detect SNPs in significant associations with AD. Results from the discovery stage in a particular dataset were then subject to further replication in the remaining three datasets. At the discovery stage, a genome-wide significance level of *p* < 5E–08 was set to select statistically significant associations, and SNPs with *p* values between 5E–08 and 5E–06 were considered suggestive AD-associated markers. These significance levels are widely accepted by genome-wide association studies in order to decrease the type I error rate (i.e., false-positive findings) due to multiple testing issues arising from investigating associations of millions of SNPs [39, 40]. A Bonferroni-corrected significance threshold of 0.0167 (i.e., 0.05/3, where 3 is the number of replication datasets for validating any significant association signals from a discovery dataset) was considered at the replication stage.

Finally, a conventional fixed-effects meta-analysis, using the inverse variance method, was conducted over the results under each plan from the four investigated datasets to obtain combined statistics for the tested SNPs. To avoid missing heterogeneous associations of opposite directions of effects, we also performed a meta-analysis on absolute values of coefficients in addition to the conventional meta-test. The results from the meta-analysis on absolute values of coefficients were used just as an additional piece of information to determine how heterogeneous effects in different cohorts can affect the results of a conventional inverse-variance meta-analysis. The meta-analysis results were interpreted according to the significance level at the discovery phase. The meta-analysis was performed using GWAMA (i.e., Genome-Wide Association Meta-Analysis) software [41].

Also, for SNPs that had significant *p* values only in males or females (i.e., plans 2 or 3), a Wald *χ*^2^ statistic with 1 degree of freedom was calculated according to the following formula [42] to investigate whether their odds ratios were significantly different between the two sexes:

$$ {\chi}^2=\frac{{\left({b}_{\mathrm{m}}-{b}_{\mathrm{f}}\right)}^2}{se_{\mathrm{m}}^2+{se}_{\mathrm{f}}^2} $$

where *b*_m_ and *b*_f_ are the coefficients (i.e., the natural logarithm of odds ratios) for any SNP in males and females, respectively, and *se*_m_ and *se*_f_ are their corresponding standard errors.

The significant findings from GWA analyses were compared to previous studies using the GRASP (i.e., Genome-Wide Repository of Associations Between SNPs and Phenotypes) search tool (v2.0.0.0) [43]. Also, LD between significant SNPs and previously detected AD-associated loci in their 1-Mb flanking regions (*r*^2^ ≥ 0.4 or significant *p* value from χ^2^ test for LD) was investigated in the CEU population (i.e., Utah Residents with Northern and Western European Ancestry) through the HaploR R package [33, 44] and the LDlink web-tool [45]. The genes coordinate’s list provided by PLINK [28] was used to find the closest genes of the significant SNPs. The chromosomal regions (i.e., cytogenetic bands) were determined using the annotation database from UCSC Genome Browser [46].

#### Gene-based analysis

Under each of three aforementioned plans, gene-based analysis was performed over the meta-analysis results using the fastBAT (i.e., Fast set-Based Association Test) method [47] implemented in the GCTA (i.e., Genome-wide Complex Trait Analysis) package (v1.26.0) [48]. This method combines *z*-statistics for a set of SNPs corresponding to each gene into a quadratic form of a multivariate normal variable. SNPs located within a gene or its 50 kb upstream/downstream regions were considered as an SNP set for that gene. The HRS dataset was used as the reference panel for LD calculation (i.e., *r*^2^ metric) in order to remove one of each pair of SNPs with *r*^2^ > 0.9 from any given set. To deal with multiple-testing issues, the false discovery rate (FDR) method suggested by Benjamini and Hochberg [49] was used to rank and select significant findings. Genes with significant *p* values at the FDR level of 0.05 were considered novel AD-associated ones if there were no SNPs with *p* < 5E–08 in their 1-Mb upstream/downstream regions in the current or previous studies.

#### Pathway-based analysis

A pathway-based analysis was also performed using the fastBAT method using the pathways predefined by the REACTOME pathway knowledgebase [50] and PID (i.e., the Pathway Interaction Database) [51]. These were provided by the molecular signatures database (MSigDB) at the Broad Institute gene set enrichment analysis (GSEA) website [52, 53]. Here, a SNP set corresponding to a particular pathway was defined as the SNPs within 50 kb of the genes in that pathway. As with the gene-based analysis, the HRS cohort was used to prune the SNP sets based on the pairwise LD measures of SNPs. The significant results were interpreted at the FDR levels of 0.05 (plans 1 and 2) and 0.025 (plan 3) to ensure that the number of possible false-positives was < 1 under each analysis plan.

#### TWA analysis

Results from conducted meta-analyses along with summary data from a publicly available expression quantitative trait loci (eQTLs) study on peripheral blood [54] were used to perform a transcriptome-wide association analysis using SMR (i.e., Summary-data-based Mendelian Randomization) software (v0.68) [55]. The eQTLs summary data were downloaded from the SMR software website. Both cis-eQTLs and trans-eQTLs were of interest. Trans-eQTLs were defined as eQTLs located at least 5 Mb away from a probe on the same chromosome or located on other chromosomes. Probes for which at least one eQTL with *p* < 5E–08 had been detected by Lloyd-Jones et al. [54] were included in our analyses provided that the corresponding eQTLs were among the genotyped or imputed SNPs in our study. This resulted in the inclusion of sets of up to 8257 probes with cis-eQTLs and 2763 probes with trans-eQTLs.

The significance of *p* values resulting from SMR testing (i.e., *P*_SMR_) was interpreted at an FDR level of 0.025–0.05. The appropriate FDR level for each of three analysis plans was chosen so we can ensure that the number of possible false-positive findings among significant probes was < 1. To identify the pleiotropic effects of SNPs on gene expression levels and AD development, probes with significant *P*_SMR_ values were then subject to heterogeneity testing (i.e., the HEIDI test) which can differentiate pleiotropy from linkage [55, 56]. Genes corresponding to probes that passed both the SMR and HEIDI tests (i.e., significant *P*_SMR_ and *P*_HEIDI_ ≥ 0.05) were deemed significant as their expression profiles might be associated with AD because of the pleiotropic effect of a single variant that affects both probe expression and AD susceptibility. Selected genes were considered potentially novel AD genes if there were no SNPs with *p* < 5E–08 within their 1-Mb upstream/downstream regions in the current or previous studies.

Finally, we also performed TWA analyses using summary results from a publically available tissue-specific eQTLs study [57] which contains eQTLs data for several regions of the brain, including the amygdala, anterior cingulate cortex (BA24), basal ganglia (e.g., caudate, nucleus accumbens, and putamen), cerebellar hemisphere, cerebellum, cortex, frontal cortex (BA9), hippocampus, hypothalamus, and substantia nigra. Once again, probes that had significant eQTLs with *p* < 5E–08 were included in our analyses. This resulted in the inclusion of sets of 597–3566 probes with cis-eQTLs (based on the brain region). The results of brain-specific TWA analyses were interpreted at a FDR level of 0.05.

### URLs

dbGaP: https://www.ncbi.nlm.nih.gov/gap; GCTA: http://cnsgenomics.com/software/gcta/#Overview; GENESIS R Package: https://bioconductor.org/packages/release/bioc/html/GENESIS.html; GRASP: https://grasp.nhlbi.nih.gov/Search.aspx; GSEA: http://software.broadinstitute.org/gsea/index.jsp; GWAMA: https://www.geenivaramu.ee/en/tools/gwama; HaploR R package: https://cran.r-project.org/web/packages/haploR/index.html; KING: http://people.virginia.edu/~wc9c/KING/; LDlink: https://ldlink.nci.nih.gov/?tab=home; LiftOver: https://genome.ucsc.edu/cgi-bin/hgLiftOver; Lme4 R Package: https://cran.r-project.org/web/packages/lme4/index.html; Minimac3: https://genome.sph.umich.edu/wiki/Minimac3; PLINK: http://zzz.bwh.harvard.edu/plink/index.shtml; SHAPEIT: https://mathgen.stats.ox.ac.uk/genetics_software/shapeit/shapeit.html; SMR: http://cnsgenomics.com/software/smr/#Overview; 1000 Genomes: http://www.internationalgenome.org/data/; https://mathgen.stats.ox.ac.uk/impute/1000GP_Phase3.html

## Results

### GWA analysis

GWA analyses were performed in four independent datasets (i.e., LOADFS, FHS, CHS, and HRS). Each of these datasets served as a discovery set to detect SNPs with significant association signals (at either a genome-wide significance level of *p* < 5E–08 or a suggestive level between 5E–08 and 5E–06), which were then subject to further replication (at the significance level of 0.0167) in the other three datasets. These analyses provided replicated and nonreplicated sets of SNPs. Finally, results from the individual datasets were combined through meta-analysis and interpreted according to the significance level at the discovery phase. Additional file 1: Tables S4–S12 provide an overview of replicated, nonreplicated, and meta-analysis sets of SNPs that were significantly associated with AD in males and females combined (plan 1) or males and females separately (plans 2 and 3). As seen in these tables, most of the newly detected AD-associated SNPs, particularly those in nonreplicated and meta-analysis sets, had significant *p* values only in one of the three study plans. For instance, among 44 and 72 newly detected SNPs in males and females, 36 and 51 SNPs had sex-specific significant *p* values, respectively. Additional file 1: Figures S1–S6 show the Manhattan and QQ plots of the GWA results in the four investigated datasets, as well as in the conducted meta-analyses under these three plans. In general, SNPs with *p* values smaller than the genome-wide significance threshold were mostly located on chromosome 19.

#### Replicated sets of SNPs

The replicated sets of SNPs under plans 1–3 contained 31, 20, and 23 SNPs, respectively (Additional file 1: Tables S4–S6). These SNPs had significant *p* values at the genome-wide level or a suggestive level of associations at the discovery stage and were then replicated in another dataset. Additional files 2, 3, and 4 contain detailed information (e.g., allele frequencies, odds ratios (ORs), *p* values, etc.) about the replicated SNPs in the four tested datasets under the three analysis plans. Notably, 12, 8, and 8 replicated SNPs, respectively, had not been previously associated with AD. The other SNPs had some evidence of direct association signals [43]. Among previously detected SNPs, rs9882471 (plan 2) was nominally associated with AD in previous studies (5E–06 ≤ *p* < 5E–02) [58].

Most of the newly detected SNPs were located inside a previously well-known susceptibility region for AD on chromosome 19q13 (i.e., *APOE* cluster gene region) and were mostly significant under different analysis plans. This subset of newly detected SNPs mostly had *p* < 5E–08, the same directions of effects in discovery and replication datasets, and significant *p* values (at genome-wide or suggestive levels of significance) in the meta-analysis.

Table 2 summarizes information about the four newly detected SNPs located outside the chromosome 19q13 region. Among these SNPs, rs62402815 was significant under plan 1 (i.e., males and females) and plan 3 (i.e., only females); and rs9918162 and rs726411 were significant only in males (i.e., plan 2). Their association signals were significant only at the suggestive level of associations (except rs62402815, which had a genome-wide level significant *p* = 1.2E–08 in females) in the discovery stage. The two SNPs that were significant in males did not have *p* < 5E–06 in conventional fixed-effects meta-analyses, which might be partially due to the heterogeneity of their effects across different datasets. These heterogeneous effects were reflected by high *i*^2^ inconsistency metrics and significant *Q*-statistics in Cochran’s heterogeneity test (*P*_q_ < 0.05). A meta-analysis based on the absolute values of the coefficients confirmed a substantial role of heterogeneity by providing smaller *p* values for most of these SNPs.

**Table 2:** Newly detected replicated and meta-analysis sets of significant SNPs located outside chromosome 19q13 region

Chromosome	Closest gene	SNP	Position	A1	Sig?	*P*_min_	Effects	Freq	OR (se)	*P*_meta_	*P*_q_	*i* ^2^	*P*_abs_	Proxy?	Gene?	Region?
Plan 1—males and females																
6p22.3	*LOC101928519*	rs62402815^a^	19,350,484	G	NYYN	1.94E–07	– – – –	0.941	0.690 (0.050)	1.80E–06	1.41E–04	0.853	1.80E–06	N	N	N
7q22.1	*TRIM56*	rs10953322	101,103,377	G	YYNN	2.89E–03	– – – –	0.871	0.758 (0.043)	4.65E–06	7.84E–01	0	4.66E–06	N	N	Y
9p22.3	C9orf92	rs4961664	16,161,235	T	YNNY	3.45E–03	– – – –	0.711	0.811 (0.035)	4.44E–06	8.14E–01	0	4.44E–06	N	N	N
9p13.2	*PAX5*	rs2282079	37,036,250	G	Y?NN	6.49E–05	– ? – –	0.959	0.595 (0.06)	3.81E–06	4.48E–01	0	3.81E–06	N	N	N
11p15.5	*AP2A2*	rs10794342	924,904	C	YNYY	2.46E–04	– – – –	0.456	0.824 (0.033)	4.45E–06	1.19E–01	0.488	4.42E–06	Y	Y	Y
13q33.3	*MYO16*	rs9555561	109,152,426	C	YNNN	6.99E–05	+ + + +	0.727	1.249 (0.056)	2.53E–06	4.64E–01	0	2.54E–06	N	Y	Y
13q33.3	*MYO16*	rs912322	109,155,938	A	YNNY	5.44E–05	+ + + +	0.733	1.260 (0.057)	1.29E–06	4.56E–01	0	1.29E–06	N	Y	Y
17q12	*LHX1*	rs8070114	36,817,647	A	NNNY	6.07E–04	– – – –	0.949	0.675 (0.053)	3.58E–06	6.06E–01	0	3.58E–06	N	N	N
17q12	*LHX1*	rs1497197	36,819,274	A	NNNY	7.69E–04	– – – –	0.949	0.668 (0.053)	2.96E–06	4.79E–01	0	2.98E–06	N	N	N
18q12.1	*MIR302F*	rs35242772	30,214,382	C	YYNY	3.31E–03	+ + + +	0.356	1.231 (0.051)	1.31E–06	8.42E–01	0	1.31E–06	N	N	Y
21q21.2	*LINC00158*	rs76252969	25,277,446	G	YYNN	1.12E–04	– – – –	0.971	0.594 (0.061)	4.86E–06	3.47E–01	0.092	4.86E–06	N	N	N
21q21.3	*LINC00515,MRPL39*	rs2298369	25,583,969	C	YYYN	6.02E–06	– – – +	0.605	0.802 (0.033)	3.67E–07	1.81E–04	0.849	1.43E–10	N	N	Y
Plan 2—only males																
5q15	*LNPEP*	rs9918162^a^	96,987,845	T	YNYN	4.66E–06	+ – – –	0.966	0.958 (0.138)	8.00E–01	2.95E–08	0.921	2.27E–06	N	N	N
8q24.22	*ADCY8*	rs726411^a^	130,734,543	G	NYNY	2.16E–06	– – + –	0.939	0.621 (0.071)	2.45E–04	5.65E–04	0.828	1.65E–05	N	Y	Y
3p14.1	*KBTBD8*	rs9862849	66,855,351	C	YNNY	1.12E–03	– – – –	0.900	0.603 (0.059)	2.66E–06	7.53E–01	0	2.65E–06	N	N	Y
23q21.31	*KLHL4*	rs5969117	87,181,248	C	YN?N	1.67E–05	+ + ? +	0.295	1.490 (0.114)	1.42E–06	8.61E–01	0	1.42E–06	N	N	Y
Plan 3—only females																
6p22.3	*LOC101928519*	rs62402815^a,b^	19,350,484	G	NYYN	1.20E–08	– – – –	0.941	0.610 (0.054)	4.29E–07	5.89E–04	0.827	4.31E–07	N	N	N
2p13.3	*ANTXR1*	rs7561207	69,138,666	A	Y??N	7.35E–05	– ? ? –	0.055	0.470 (0.066)	4.08E–06	9.22E–01	0	4.08E–06	N	N	N
4p16.2	*STK32B*	rs17675640	5,095,813	G	YYYN	7.90E–04	– – – –	0.667	0.769 (0.040)	1.89E–06	4.52E–01	0	1.89E–06	N	Y	Y
4p16.2	*STK32B*	rs6838792	5,096,839	C	YYYN	7.04E–05	– – – –	0.617	0.772 (0.040)	1.61E–06	1.17E–01	0.491	1.61E–06	N	Y	Y
4p16.2	*STK32B*	rs895681	5,099,404	T	YYYN	1.23E–04	– – – –	0.617	0.776 (0.040)	2.72E–06	1.41E–01	0.450	2.72E–06	N	Y	Y
6p21.33	*TNXB*	rs11969759	32,053,353	C	NNYY	3.65E–04	– – – –	0.942	0.616 (0.057)	2.10E–06	3.86E–01	0.012	2.10E–06	N	N	Y
6p21.33	*TNXB*	rs10947230	32,056,618	C	NNYY	3.19E–04	– – – –	0.941	0.619 (0.057)	2.33E–06	4.36E–01	0	2.34E–06	N	N	Y
6p21.33	*TNXB*	rs7774197	32,078,498	A	NNYY	3.68E–04	– – – –	0.941	0.627 (0.058)	4.17E–06	3.47E–01	0.092	4.20E–06	N	N	Y
9q22.2	*SYK*	rs1172922	90,726,252	A	NYNY	5.27E–04	+ + + +	0.118	1.408 (0.098)	4.56E–06	1.14E–01	0.495	4.46E–06	N	N	N
12q24.33	*SFSWAP*	rs73156187	131,542,412	G	YNYN	1.35E–04	– – – –	0.894	0.695 (0.051)	4.47E–06	2.08E–01	0.341	4.45E–06	N	N	Y
12q24.33	*SFSWAP*	rs7963314	131,573,284	G	YNYN	6.65E–04	– – – –	0.881	0.700 (0.049)	2.72E–06	4.06E–01	0	2.73E–06	N	N	Y
21q21.3	*MIR155HG*	rs12386284^b^	25,517,756	T	YYN?	3.61E–03	+ + + ?	0.249	1.367 (0.087)	4.55E–06	9.24E–01	0	4.54E–06	N	N	Y
21q21.3	*MIR155HG*	rs1783012	25,547,104	T	YYN?	2.11E–03	– – – ?	0.748	0.732 (0.047)	4.59E–06	9.40E–01	0	4.59E–06	N	N	Y
21q21.3	*MIR155HG*	rs1783013	25,547,257	T	YYN?	2.11E–03	– – – ?	0.748	0.733 (0.047)	4.65E–06	9.49E–01	0	4.65E–06	N	N	Y
21q21.3	*MIR155HG*	rs926963	25,547,744	T	YYN?	2.11E–03	– – – ?	0.748	0.732 (0.047)	4.75E–06	9.49E–01	0	4.75E–06	N	N	Y
21q21.3	*MIR155HG*	rs1893650	25,568,503	T	YYNN	2.32E–03	+ + + +	0.247	1.320 (0.075)	3.94E–06	5.41E–01	0	3.99E–06	N	N	Y
21q21.3	*MIR155HG*	rs2226326	25,569,648	A	YYNN	2.57E–03	+ + + +	0.245	1.319 (0.075)	4.38E–06	6.34E–01	0	4.38E–06	N	N	Y
21q21.3	*MIR155HG*	rs2829803^b^	25,575,998	G	YYNN	2.39E–03	+ + + +	0.247	1.319 (0.075)	4.09E–06	5.40E–01	0	4.10E–06	N	N	Y
21q21.3	*LINC00515,MRPL39*	rs2298369^b^	25,583,969	C	YNYN	6.79E–05	– – – +	0.606	0.754 (0.039)	3.11E–07	1.77E–02	0.703	2.21E–07	N	N	Y
21q21.3	*MRPL39*	rs2829823	25,599,076	A	YYNN	2.37E–03	+ + + +	0.245	1.320 (0.075)	4.06E–06	5.96E–01	0	4.07E–06	N	N	Y
21q21.3	*MRPL39*	rs2829832	25,601,939	T	YYNN	2.47E–03	+ + + +	0.245	1.318 (0.075)	4.68E–06	6.04E–01	0	4.65E–06	N	N	Y

*SNP* single-nucleotide polymorphism, *Chromosome* chromosomal region based on cytogenetic bands, *Position* position of SNP based on Human Genome version 38 (hg38), *A1* effect allele, *Sig?* if SNP had *p* < 0.0167 in LOADFS, FHS, CHS, and HRS datasets, respectively (Yes, No, Missing), P_*min*_ minimum *p* value detected for SNP in aforementioned datasets, *Effects* direction of SNP’s effects in aforementioned datasets (Positive, Negative, Missing), *Freq* frequency of effect allele based on meta-analysis, *OR (se)* odds ratio and its standard error based on meta-analysis, P_*meta*_ *p* value of SNP in meta-analysis, P_*q*_ *p* value of *Q*-statistics (Cochran’s heterogeneity test), i^*2*^, inconsistency metric, *P*_*abs*_ *p* value of SNP in meta-analysis on absolute values of effect sizes (i.e., *β* coefficients), *Proxy?* if SNP is in linkage disequilibrium with any previously detected AD-associated loci whose *p* value is less than that detected in this study (Yes, No), *Gene?* if previous studies have detected AD-associated SNPs with *p* < 5E–06 in closest gene to detected SNP (Yes, No), *Region?* if previous studies have detected AD-associated SNPs with *p* < 5E–06 in chromosomal region in which detected SNP is located (Yes, No), *Y* yes, *N* no, *AD* Alzheimer’s disease

^a^Replicated SNPs

^b^SNP did not have significant sex-specific effects. All SNPs that were significant only in males or females also had significant sex-specific effects except rs62402815, rs12386284, rs2829803, and rs2298369

Also, rs62402815 and rs726411 had the same direction of effects in the discovery and replication datasets. The directions of effects of rs9918162 were opposite in the discovery and replication sets. While genetic variants that have the same direction of effects in multiple independent cohorts are generally of more interest, those with opposite effects can be important as well because they may be indicative of the genetic heterogeneity of the studied trait in different cohorts arising, for example, from the epistasis or differences in LD patterns [59–61].

Although no evidence of direct association with AD was found in previous studies for the newly detected subsets of replicated SNPs, their 1-Mb upstream/downstream regions harbor AD-associated SNPs. We therefore investigated their LD with AD-associated loci in their 1-Mb flanking regions in the CEU population [45]. Newly detected SNPs were considered informative AD markers if their *p* values were smaller than those of the top AD-associated SNPs in their neighborhood or they were not in LD with previously AD-associated loci whose *p* values were smaller than those detected in this study. Additional file 1: Table S13 contains LD information about those newly detected SNPs for which proxy AD-associated loci have been reported. As seen in Additional file 1: Table S13, all newly detected SNPs on chromosome 19q13 had larger *p* values than the top AD-associated loci in their neighborhood and were in LD with them. Therefore, they were likely to relay the same information as their neighboring AD-associated SNPs.

On the other hand, the *p* values of SNPs located outside the chromosome 19q13 region were mostly smaller than the previously detected association signals in their flanking regions and were not in LD with such loci. As seen in Table 2, among the closest genes to these SNPs, only *ADCY8* (corresponding to rs726411 located in the 8q24.22 region) was associated with AD in previous GWAS at a suggestive level of associations (rs263238 with *p* = 2.40E–06 [62]). In addition, none of the chromosomal regions (i.e., cytogenetic bands) in which other SNPs are located contained any previously AD-associated SNPs with *p* < 5E–06 [43]. Detailed information about the genes and chromosomal regions corresponding to the newly detected SNPs that contain previously AD-associated SNPs can be found in Additional files 2, 3, and 4.

#### Nonreplicated sets of SNPs

Additional file 1: Tables S7–S9 (corresponding to plans 1–3) show that 54, 40, and 46 SNPs had significant *p* values at genome-wide or suggestive levels of associations in only one of the four datasets of interest. Most of them were newly detected (41, 33, and 40 SNPs, respectively), as there was no evidence of their direct association with AD in previous studies [43]. Also, they were mostly plan specific and demonstrated evidence of sex disparity. Most were located in chromosomal regions other than 19q13 and were significant at a suggestive level of associations. Detailed information about nonreplicated sets of SNPs (e.g., allele frequencies, ORs, *p* values, etc.) can be found in Additional files 2, 3, and 4. Of those SNPs previously associated with AD, rs11038106, rs9597722, rs723804, rs17697225 [63], rs2065706 [64] (plan 1), rs4679840 [58] (plan 2), and rs1359176 [65] (plan 3) were only nominally significant (5E–06 ≤ *p* < 5E–02) in previous studies. Once again, SNPs located outside the chromosome 19q13 region either had smaller *p* values than previously detected AD-associated loci in their proximity or were not in LD with them, except for rs34779859 on chromosome 2 (plan 3) which was significant in females. LD information about those newly detected SNPs for which proxy AD-associated loci have been previously identified can be found in Additional file 1: Table S13.

Among the closest genes to newly detected SNPs outside the chromosome 19q13 region, *BIN1*, *FRMD4A*, and *CDH4* that were significant under plan 3 were previously associated with AD with *p* < 5E–06 (rs744373 with *p* = 2.60E–14 [66], rs7921545 with *p* = 5.40E–07 [67], and rs4925189 with *p* = 6.30E–07 [68], respectively). Also, several other genes were located in AD-associated chromosomal regions. Information about these genes/regions is summarized in Additional files 2, 3, and 4.

#### Meta-analysis sets of SNPs

Additional file 1: Tables S10–S12 show that 17, 4, and 24 SNPs that were not among replicated or nonreplicated sets of significant SNPs under analysis plans 1–3 passed the significance threshold in the meta-analysis. Additional files 2, 3, and 4 summarize the GWA results for these SNPs. The meta-analysis *p* values of these SNPs were mostly significant at the level of suggestive associations, except for rs76366838, rs115881343 (plan 1), rs73048293, rs57537848, and rs76366838 (plan 3) on chromosome 19q13 which had *p* < 5E–08. Also, they were mostly located outside chromosome 19q13 and were plan specific (i.e., they were not among replicated, nonreplicated, or meta-analysis sets of significant SNPs under other plans). For example, significant SNPs in males were not significant in females and vice versa. In addition, most SNPs (14, 3, and 24 SNPS under plans 1–3, respectively) were not associated with AD in previous studies [43]. Summary information about the newly detected subset of meta-analysis sets of SNPs that were outside chromosome 19q13 is presented in Table 2. As with the replicated and nonreplicated sets of SNPs, most of the newly detected SNPs not on chromosome 19q13 had smaller *p* values than the ones reported for their nearby AD-associated loci or were not in LD with them. These SNPs, therefore, were considered novel and informative AD markers. On the other hand, proxy AD-associated SNPs were found for all newly detected SNPs that were located on chromosome 19q13 (Additional file 1: Table S13).

As seen in Table 2, among the closest genes to newly detected SNPs outside the chromosome 19q13 region, *AP2A2* (corresponding to rs10794342), *MYO16* (corresponding to rs9555561 and rs912322 in the 13q33.3 region) and *STK32B* (corresponding to rs17675640, rs6838792, and rs895681 in the 4p16.2 region) were previously associated with AD with *p* < 5E–06 (rs17393344 with *p* = 1.70E–08; and rs78647349 with *p* = 5.20E–07, respectively [69]). In addition, several chromosomal regions including 3p14.1 (*KBTBD8*), 6p21.33 (*TNXB*), 7q22.1 (*TRIM56*), 12q24.33 (*SFSWAP*), 18q12.1 (*MIR302F*), 21q21.3 (*MIR155HG*, *LINC00515*, and *MRPL39*), and 23q21.31 (*KLHL4*) were associated with AD at a suggestive level of associations by previous GWAS. However, no AD-associated SNPs with *p* < 5E–06 have been previously detected in chromosomal regions corresponding to *C9orf92*, *PAX5*, *LHX1*, and *LINC00158* genes (i.e., 9p22.3, 9p13.2, 17q12, and 21q21.2, respectively) that were significant under plan 1; and *ANTXR1* and *SYK* genes (i.e., 2p13.3 and 9q22.2, respectively) [43]. Detailed information about these AD-associated genes and chromosomal regions is provided in Additional files 2, 3, and 4.

#### Nominally significant sets of SNPs

Under each of the three analysis plans, there were several SNPs associated with AD at a nominal level of significance (5E–06 ≤ *p* < 5E–02) in all datasets they were present in. They were mostly present in three datasets as they were excluded from one dataset by the QC procedure. These SNPs (30, 28, and 28 SNPs under plans 1–3, respectively) are listed in Additional files 2, 3, and 4. Although they did not have highly significant *p* values, they are reported here due to the consistency in their association signals that was observed in multiple tested datasets. With the exception of rs575088, which had nominally significant *p* values in all datasets under plans 1 and 3, the significance pattern of the other SNPs was observed under only one plan. Also, rs2282079 (detected in females) was among the meta-analysis set of SNPs under plan 1 as well. None of these SNPs had *p* < 5E–06 in the conducted meta-analyses. The lack of meta-analysis power could be due to the small sample size, weak association signals, absence of some SNPs in one dataset, or heterogeneous effects of some SNPs across the different datasets as evidenced by their high *i*^2^ values, significant *Q* tests, and smaller *p* values in meta-analysis on absolute values of coefficients. The SNPs whose associated signals were reported here for the first time were not in LD with previously detected AD-associated loci (*p* < 5E–06) in their 1-Mb flanking regions (Additional file 1: Table S13). Interestingly, 22 out of 28 SNPs detected in males had the opposite pattern of significance in females (i.e., *p* > 0.05 in all datasets). Also, 26 out of 28 SNPs detected in females had the opposite pattern of significance in males (Additional file 5). Not all SNPs with opposite patterns of significance in females-only vs males-only analyses had the same pattern in the meta-analysis. Closest genes to some of these SNPs were located in chromosomal regions that were previously associated with AD with *p* < 5E–06. Information about these genes/regions can be found in Additional files 2, 3, and 4.

#### Adjustment by APOE SNPs

For the AD-associated SNPs that were located on chromosome 19, we further investigated whether their association signals may change after adjustment for *APOE* genotypes in the models. For each subject, the *APOE* genotype was determined based on its genotypes at rs429358 and rs7412 loci using the coding schema provided in Additional file 1: Table S14. We found that none of the tested SNPs had *p* < 5E–06 once *APOE* was added as a covariate to the models.

Additional file 1: Table S15 summarizes the information regarding the LD between SNPs detected in our study and *APOE* SNPs. Among newly AD-associated SNPs, only six SNPs were in LD with one or both of the *APOE* SNPs. Others were not in LD with the two *APOE* SNPs (i.e., *r*^2^ = 0.001–0.072) [45]. Therefore, it should be noted that despite a major impact of the *APOE* genotypes on the associations of other SNPs inside the chromosome 19q13 region with AD, this result would not automatically imply that the *APOE* SNPs (i.e., rs429358 and rs7412) are the only contributors to AD pathogenesis because *APOE*-adjusted models highlighted the statistical correlations rather than biological (i.e., genetic) linkage. Further analyses such as those examining the role of haplotypes and epistatic interactions would be helpful to more comprehensively dissect the genetic heterogeneity of this region, and to elucidate the biological relevance of the *APOE*-adjusted models [70].

#### Sex-specific effects

We also investigated the sex-specific effects of SNPs that were significantly associated with AD only in males or females by performing a Wald χ^2^ test to determine whether their odds ratios were significantly different between males and females. Additional file 1: Tables S16 and S17 summarize the results from this test for replicated, nonreplicated, and meta-analysis sets of AD-associated SNPs. We found that the differences between odds ratios of the SNPs in males and females were significant (*p* < 0.05) in most cases, except rs62405605, rs1062851, rs62510850, rs7000333, rs6572843 (among nonreplicated set of SNPs in females), and rs12386284 (among meta-analysis set of SNPs in females). Detailed information about the results from the Wald χ^2^ test can be found in Additional file 6.

In addition, the SNPs that had significant *p* values only in males or females were searched against the GRASP catalog [43] to find out whether they were among the known sex-linked autosomal SNPs or were associated with any other diseases/traits at suggestive level of associations. We noticed that there was no evidence of such associations in previous studies.

### Gene-based analysis

The significant findings from gene-based analyses corresponding to plans 1–3 are summarized in Table 3. Under all plans, most genes with significant *p* values at the FDR of 0.05 were located in the chromosome 19q13 region. Since the chromosome 19q13 region harbors several SNPs with *p* < 5E–08 in both current and previous studies, significant genes in this region are not discussed here as they do not meet the criteria set for detecting novel AD genes. The only significant genes outside the *APOE* cluster region were *LINC00158* under plan 1 and *LINC00158*, *MIR155HG*, *MIR155*, *LINC00515*, *MRPL39*, and *JAM2* under plan 3 that were located in the chromosome 21q21.3 region. None of the SNPs inside or within 1-Mb flanking regions of these genes had significant *p* values at the genome-wide level in our study, although several had suggestive-level *p* values in conducted meta-analyses under plans 1 and 3. Also, SNPs in 1-Mb nearby regions of these genes were only nominally associated with AD (8.0E–04 < *p* < 5E–02) in previous studies [58, 65, 71–73]. However, the chromosome 21q21.3 region was associated with AD by previous GWAS at a suggestive level of associations (rs239713 with *p* = 5.00E–07 [68]). This SNP is located ~ 1.6 Mb away from significant genes reported in our study.

**Table 3:** Significantly AD-associated genes from gene-based analyses

Gene	Chromosomal region	Start	End	Number of SNPs	Plan 1 (males and females)		Plan 2 (only males)		Plan 3 (only females)	
					*χ* ^2^	*p* value	*χ* ^2^	*p* value	*χ* ^2^	*p* value
*CEACAM16*	19q13.32	44,699,150	44,710,714	83	330.47	7.52E–06	NS	NS	NS	NS
*BCL3*	19q13.32	44,748,720	44,760,044	63	274.81	3.95E–06	NS	NS	254.31	5.44E–06
*MIR8085*	19q13.32	44,758,656	44,758,721	52	235.68	8.72E–06	NS	NS	NS	NS
*CBLC*	19q13.32	44,777,868	44,800,646	62	419.91	2.02E–10	NS	NS	NS	NS
*BCAM*	19q13.32	44,809,058	44,821,421	51	455.39	1.07E–10	NS	NS	298.41	5.00E–07
*PVRL2*	19q13.32	44,846,135	44,889,228	90	3861.00	4.74E–72	1357.12	4.52E–25	2304.33	7.11E–43
*TOMM40*	19q13.32	44,891,219	44,903,689	73	3664.74	9.12E–86	1288.14	8.98E–30	2171.92	1.26E–50
*APOE*	19q13.32	44,905,781	44,909,393	70	3590.18	4.32E–85	1262.22	1.09E–29	2121.68	3.58E–50
*APOC1*	19q13.32	44,914,663	44,919,349	67	3482.70	4.76E–87	1230.83	1.63E–30	2049.54	4.25E–51
*APOC1P1*	19q13.32	44,926,802	44,931,386	57	3427.13	6.60E–93	1209.89	1.01E–32	2008.34	2.00E–54
*APOC4*	19q13.32	44,942,237	44,945,496	46	3063.64	5.63E–89	1058.60	7.90E–31	1792.11	3.94E–52
*APOC4–APOC2*	19q13.32	44,942,237	44,949,565	46	3063.64	5.63E–89	1058.60	7.90E–31	1792.11	3.94E–52
*APOC2*	19q13.32	44,945,981	44,949,566	42	2242.85	8.65E–73	760.49	1.16E–24	1347.91	9.14E–44
*CLPTM1*	19q13.32	44,954,584	44,993,346	47	2037.76	3.42E–60	700.13	9.56E–21	1217.18	5.03E–36
*LINC00158*	21q21.3	25,385,819	25,431,701	39	223.58	1.20E–06	NS	NS	181.03	2.27E–05
*MIR155HG*	21q21.3	25,562,144	25,575,168	27	NS	NS	NS	NS	170.35	1.52E–05
*MIR155*	21q21.3	25,573,979	25,574,044	22	NS	NS	NS	NS	152.69	7.00E–06
*LINC00515*	21q21.3	25,582,774	25,583,224	24	NS	NS	NS	NS	156.49	9.36E–06
*MRPL39*	21q21.3	25,585,655	25,607,489	36	NS	NS	NS	NS	269.62	4.20E–06
*JAM2*	21q21.3	25,639,281	25,717,562	53	NS	NS	NS	NS	276.76	3.02E–05

Genomic coordinates based on Human Genome version 38 (hg38)

*AD* Alzheimer’s disease, *SNP* single-nucleotide polymorphism, *NS* nonsignificant

### Pathway-based analysis

We found that 19, 10, and 19 pathways were significantly associated with AD under plans 1–3, respectively (Table 4). The proper FDR levels at which the numbers of possible false-positives were less than 1 were 0.05 under plans 1 and 2, and 0.025 under plan 3. We found that 12 pathways were significant under two or three analysis plans (i.e., they were not plan specific). There were also seven pathways that were significant only under plan 1 (males and females), and seven others were significant only in females (i.e., plan 3). No pathways were specifically significant in males (i.e., plan 2).

**Table 4:** Significantly AD-associated pathways from pathway-based analyses

Pathway	Pathway GSEA ID	Number of genes	Plan 1 (males and females)		Plan 2 (only males)		Plan 3 (only females)	
			*χ* ^2^	*p* value	*χ* ^2^	*p* value	*χ* ^2^	*p* value
Mitochondrial protein import	M590	58	4939.52	3.83E–44	2741.07	4.65E–12	3622.61	7.07E–25
Chylomicron-mediated lipid transport	M14162	16	4180.44	1.11E–41	1968.73	7.13E–14	2854.57	4.86E–25
Nectin adhesion pathway^a^	M72	30	5872.18	6.52E–39	3393.55	6.09E–09	4310.19	1.39E–19
HDL-mediated lipid transport	M5056	15	4184.21	5.08E–38	2195.67	1.98E–12	2779.67	6.00E–20
Lipoprotein metabolism	M3462	28	5221.10	2.11E–37	3011.78	1.86E–10	3825.74	3.47E–20
Lipid digestion, mobilization and transport	M1023	46	5889.33	5.64E–36	3631.17	4.67E–09	4448.01	2.37E–18
E-cadherin stabilization pathway^a^	M232	42	5976.76	1.72E–27	3670.50	9.69E–05	4517.86	3.67E–12
Immunoregulatory interactions between a lymphoid and a nonlymphoid cell	M8240	70	6335.34	4.04E–19	4056.72	2.39E–07	4720.40	8.48E–11
Adherens junctions interactions	M11980	27	7432.11	7.54E–18	5204.26	1.54E–05	6110.31	1.67E–10
Cell–cell junction organization	M820	56	8940.07	1.34E–17	6464.13	2.61E–04	7758.51	4.48E–11
Cell junction organization	M19248	78	10,542.40	1.71E–17	NS	NS	9337.94	6.78E–11
NF-κB atypical pathway^a^	M26	17	1630.19	3.26E–07	NS	NS	NS	NS
Platelet sensitization by LDL	M919	16	994.69	5.38E–04	NS	NS	1002.45	4.21E–04
Signaling by EGFR in cancer	M563	109	8116.41	6.44E–04	NS	NS	NS	NS
Trans-Golgi network vesicle budding	M539	60	2967.31	7.13E–04	NS	NS	NS	NS
FAS (CD95) signaling pathway^a^	M94	38	2322.57	7.15E–04	NS	NS	NS	NS
Golgi-associated vesicle biogenesis	M1877	53	2758.56	7.26E–04	NS	NS	NS	NS
Signaling by PDGF	M2049	122	9443.31	7.57E–04	NS	NS	NS	NS
mRNA processing	M2531	161	6183.56	7.93E–04	NS	NS	NS	NS
Prostacyclin signaling through prostacyclin receptor	M926	19	NS	NS	NS	NS	1579.24	4.50E–05
G-protein activation	M13115	27	NS	NS	NS	NS	2138.82	6.18E–05
ADP signaling through P2Y purinoceptor 12	M841	21	NS	NS	NS	NS	1680.25	1.54E–04
ADP signaling through P2Y purinoceptor 1	M811	25	NS	NS	NS	NS	2101.01	2.71E–04
Glucagon-type ligand receptors	M10322	33	NS	NS	NS	NS	2483.81	4.51E–04
Signal amplification	M9379	31	NS	NS	NS	NS	2412.57	4.99E–04
Gβγ signaling through PI3Kγ	M14301	25	NS	NS	NS	NS	1808.24	5.30E–04

*AD* Alzheimer’s disease, *GSEA* Gene Set Enrichment Analysis Platform, *HDL* high-density lipoprotein, *LDL* low-density lipoprotein, *EGFR* epidermal growth factor receptor, *PDGF* platelet-derived growth factor, *NS* nonsignificant

^a^Definition of pathway is based on the Pathway Interaction Database (PID). Other pathways are from the REACTOME pathway knowledgebase

### TWA analysis

#### Analyzing probes with cis-eQTLs

Using eQTLs data from peripheral blood, we found that four, eight, and four probes/genes passed both the SMR (*P*_SMR_ < 6.03E–05) and HEIDI (*P*_HEIDI_ ≥ 0.05) tests under plans 1–3, respectively. The significant FDR level for interpreting the results from the SMR test was set to 0.05 under plan 1 and 0.025 under plans 2 and 3 to ensure that the expected number of false-positive findings was < 1. Table 5 presents information about these 16 probes/genes, their top eQTLs, and the respective *p* values. The top eQTLs corresponding to these probes/genes were all nominally significant in our GWA analyses (2.01E–04 ≤ *P*_GWAS_ ≤ 2.47E–02). Moreover, we did not identify any SNPs with significant *p* values at the genome-wide significance level within 1 Mb of these genes. However, several SNPs within 1 Mb of *MS4A6A* [64, 66, 74, 75] and *UQCC* [76] were associated with AD with *p* < 5E–08 in previous studies. Among 14 other genes, SNPs in regions around *TRA2A* [64], *IRAK3* [77], and *ESPN* [78] were previously associated with AD at the suggestive level of associations. In addition, *ATG10* [77] and *LPXN* [74] were located in chromosomal regions (i.e., 5q14.1 and 11q12.1) that contained AD-associated SNPs with *p* < 5E–06.

**Table 5:** Significantly AD-associated probes/genes from transcriptome-wide analyses

Probe ID	Gene	Top eQTL	Chromosomal region	Position	A1	A1 Freq	*P*_GWAS_	*P*_eQTL_	*b*_SMR_	SE_SMR_	*P*_SMR_	*P*_HEIDI_
Plan 1—males and females												
ILMN_1754501	*C2orf74*	rs720201	2p15	61,149,328	C	0.413	6.94E–04	1.34E–164	0.189	0.047	5.54E–05	6.64E–01
ILMN_2206098	*ATG10*	rs11741569	5q14.1	82,030,091	G	0.090	9.96E–04	9.69E–27	−0.391	0.097	5.66E–05	8.32E–02
ILMN_2359800	*MS4A6A*	rs7108663	11q12.2	60,260,669	C	0.412	2.17E–04	1.41E–48	0.380	0.088	1.59E–05	8.57E–01
ILMN_2343047	*ABCB9*	rs641760	12q24.31	123,034,319	T	0.214	2.75E–04	9.79E–17	−0.650	0.162	6.03E–05	1.14E–01
Plan 2—only males												
ILMN_1700307	*ZNF815*	rs117856560	7p22.1	5,852,503	C	0.048	1.50E–02	8.23E–49	0.389	0.092	2.22E–05	8.80E–02
ILMN_1731043	*TRA2A*	rs1046135	7p15.3	23,530,965	T	0.135	1.89E–03	4.32E–115	−0.294	0.067	1.07E–05	2.51E–01
ILMN_1671603	*MED30*	rs10111328	8q24.11	117,525,450	T	0.426	3.79E–04	1.67E–20	1.011	0.232	1.30E–05	8.22E–02
ILMN_1742789	*LPXN*	rs7928565	11q12.1	58,587,146	C	0.012	2.47E–02	7.17E–86	−0.287	0.056	2.68E–07	6.20E–01
ILMN_1913678	*IRAK3*	rs1436849	12q14.3	66,181,338	T	0.416	1.25E–03	6.14E–35	−0.656	0.160	4.11E–05	6.60E–02
ILMN_1737561	*N4BP2L2*	rs718444	13q13.1	32,572,915	T	0.375	1.16E–03	1.80E–53	−0.489	0.119	3.96E–05	2.90E–01
ILMN_1724734	*UQCC*	rs2425062	20q11.22	35,335,763	G	0.383	2.01E–04	4.58E–13	−1.356	0.321	2.42E–05	7.78E–01
ILMN_2296950	*APOBEC3F*	rs11089928	22q13.1	39,133,424	A	0.068	2.42E–03	7.21E–20	−0.724	0.168	1.60E–05	8.31E–02
ILMN_1806607	*SFN* ^*a*^	rs3817604	1p36.11/4p16.3	1,297,549	T	0.120	4.10E–04	5.77E–10	1.179	0.292	5.49E–05	3.92E–01
Plan 3—only females												
ILMN_1806710	*ESPN*	rs12074379	1p36.31	6,434,683	T	0.040	7.26E–03	8.51E–40	0.421	0.101	3.38E–05	2.84E–01
ILMN_1696003	*GNAI3*	rs2301229	1p13.3	109,594,056	T	0.175	4.92E–04	1.45E–211	−0.209	0.045	4.00E–06	1.21E–01
ILMN_1741881	*C9orf72*	rs2282240	9p21.2	27,572,636	T	0.264	2.49E–04	1.18E–90	−0.327	0.070	3.27E–06	5.39E–02
ILMN_1803925	*MTMR3*	rs41174	22q12.2	30,030,080	T	0.325	8.34E–04	3.03E–76	0.321	0.078	4.21E–05	4.12E–01

Genomic coordinates are based on Human Genome version 38 (hg38)

*AD* Alzheimer’s disease, *eQTL* expression quantitative trait loci, *A1* effect allele, *A1/A1 freq* effect allele and its frequency, *GWAS* genome-wide association study, *SMR* summary-data-based Mendelian randomization, *SE* standard error, *HEIDI* Heterogeneity in Dependent Instruments

^a^Trans-eQTL

Our TWA analyses on brain-specific eQTLs data revealed associations of two probes/genes with AD in males (i.e., *CRIPAK* and *PRDM10*), and two others in females (i.e., *AHSA2* and *ATG10*) at the FDR level of 0.05 (Table 6). No probe/gene passed the SMR and HEIDI tests under analysis plan 1. The probe corresponding to the *AHSA2* gene was significantly associated with AD in several brain regions (i.e., caudate basal ganglia, cerebellum, cortex, hypothalamus, nucleus accumbens, putamen basal ganglia, and substantia nigra). Also, *ATG10* was significantly associated with AD in the nucleus accumbens and putamen basal ganglia. The corresponding top eQTLs were nominally significant in our GWA analyses in males and females (4.30E–05 ≤ *P*_GWAS_ ≤ 9.33E–02). There were no SNPs with significant *p* values at the genome-wide significance level within 1 Mb of these genes in our study; however, SNPs with significant *p* values at the suggestive level of significance were found in flanking regions of *ATG10* in the nonreplicated set of SNPs in females (see Additional file 4). In addition, the SNPs within 1 Mb of these four genes were only nominally associated with AD in previous studies [43]. In terms of chromosomal regions, in addition to *ATG10* as explained earlier, SNPs in the chromosome 11q24.3 region (*PRDM10* gene) were also previously associated with AD at a genome-wide significance level [69].

**Table 6:** Significantly AD-associated probe/genes from transcriptome-wide analyses on brain tissue data

Probe ID	Gene	Top eQTL	Chromosomal region	Position	A1	A1 Freq	*P*_GWAS_	*P*_eQTL_	*b*_SMR_	SE_SMR_	*P*_SMR_	*P*_HEIDI_	Brain region
Plan 2—only males													
ENSG00000179979.7	*CRIPAK*	rs3817604	4p16.3	1,297,549	T	0.120	4.10E–04	1.14E–09	0.358	0.089	6.24E–05	4.13E–01	Hippocampus
ENSG00000170325.10	*PRDM10*	rs55746218	11q24.3	129,964,624	C	0.076	9.33E–03	2.10E–09	0.867	0.205	2.33E–05	2.81E–01	Cerebellar hemisphere
Plan 3—only females													
ENSG00000173209.18	*AHSA2*	rs2600667	2p15	61,177,796	T	0.297	1.75E–04	1.53E–22	−0.330	0.075	1.19E–05	9.70E–02	Caudate basal ganglia
ENSG00000173209.18	*AHSA2*	rs2600667	2p15	61,177,796	T	0.297	1.75E–04	9.79E–40	−0.216	0.047	4.36E–06	1.29E–01	Cerebellum
ENSG00000173209.18	*AHSA2*	rs2600667	2p15	61,177,796	T	0.297	1.75E–04	6.30E–28	−0.292	0.065	7.72E–06	1.33E–01	Cortex
ENSG00000173209.18	*AHSA2*	rs2600667	2p15	61,177,796	T	0.297	1.75E–04	2.30E–15	−0.335	0.080	3.08E–05	8.00E–02	Hypothalamus
ENSG00000173209.18	*AHSA2*	rs2600667	2p15	61,177,796	T	0.297	1.75E–04	2.65E–24	−0.262	0.059	1.01E–05	7.39E–02	Nucleus accumbens
ENSG00000173209.18	*AHSA2*	rs2600667	2p15	61,177,796	T	0.297	1.75E–04	7.23E–22	−0.217	0.050	1.27E–05	2.11E–01	Putamen basal ganglia
ENSG00000173209.18	*AHSA2*	rs2600667	2p15	61,177,796	T	0.297	1.75E–04	5.43E–13	−0.331	0.082	5.05E–05	3.20E–01	Substantia nigra
ENSG00000152348.11	*ATG10*	rs11748868	5q14.1	82,066,661	C	0.091	4.30E–05	2.70E–09	−0.281	0.066	1.85E–05	8.05E–02	Nucleus accumbens
ENSG00000152348.11	*ATG10*	rs11748868	5q14.1	82,066,661	C	0.091	4.30E–05	1.39E–09	−0.263	0.061	1.55E–05	1.02E–01	Putamen basal ganglia

Genomic coordinates are based on Human Genome version 38 (hg38)

*AD* Alzheimer’s disease, *eQTL* expression quantitative trait loci, *A1* effect allele, *A1/A1 freq* effect allele and its frequency, *GWAS* genome-wide association study, *SMR* summary-data-based Mendelian randomization, *SE* standard error, *HEIDI* Heterogeneity in Dependent Instruments

#### Analyzing probes with trans-eQTLs

Using eQTLs data from peripheral blood, one probe mapping to the *SFN* gene on chromosome 1p36 had significant *P*_SMR_ at the FDR level of 0.05, and passed the HEIDI test under plan 2 (Table 5). The corresponding top eQTL was located on chromosome 4p16 in the intronic region of the *MAEA* gene and was nominally associated with AD in our study (*P*_GWAS_ = 4.10E–04). There were no significant association signals at the genome-wide significance level in the *SFN* gene or its 1-Mb flanking regions in current or previous studies [43].

## Discussion

The genetic architecture of AD has been widely studied in recent years, and so far more than 60,000 SNPs have been associated with AD with *p* < 0.05. Of these, 281 SNPs (mapped to 49 genes) and 593 SNPs (mapped to 165 genes) had significant *p* values at the genome-wide and suggestive levels of associations, respectively [43]. Despite these efforts, a major proportion of *h*^2^ of AD has remained unexplained. Exploring the genetic risk factors contributing to AD is highly important from a precision medicine perspective where the goal is to personalize diagnostic and therapeutic interventions. The current study provides further insight into the genetic architecture of AD through GWA, TWA, gene-based, and pathway-based analyses of four independent datasets. These datasets, particularly the LOADFS cohort, were partially used in previous genetic studies of AD [21, 72, 75, 79–87].

Our GWA analyses corroborated the associations of a number of previously detected AD loci and revealed some significant novel association signals. Among previously detected AD-associated SNPs, we found several SNPs with *p* values that were smaller than those reported before. Also, the significant association signals for three SNPs inside the chromosome 19q13 region (i.e., nonreplicated rs2965169 SNP under plan 1, rs10426423 from the meta-analysis set of SNPs under plan 1, and rs769450 from the replicated set of SNPs under plan 1 and the nonreplicated sets of SNPs under plans 2 and 3) were previously reported only in African-Americans (*p* = 2.6E–8, *p* = 9.9E–7, and *p* = 5.3E–27, respectively [88]). Most newly detected AD-associated SNPs, particularly those outside the chromosome 19q13 region, can be considered informative AD markers because their *p* values in our study were smaller than those for other AD-associated loci in their 1-Mb upstream/downstream regions and they were not in LD with such loci. For instance, as seen in Table 2 that summarizes the replicated and meta-analysis sets of SNPs, 11, 4, and 21 novel AD-associated SNPs were detected under plans 1–3, respectively. These SNPs were mapped to 21 genes in 19 chromosomal regions (i.e., cytogenetic regions). Of these, four genes had been associated with AD in previous GWAS with *p* < 5E–06. Also, nine genes were located in eight chromosomal regions that contained previously AD-associated SNPs that were > 1 Mb away from the SNPs detected in our study. The other eight genes/regions had not been associated with AD in previous studies at genome-wide or suggestive levels of associations [43].

Our GWA analyses also revealed associations of a number of SNPs (41, 33, and 40 SNPs under plans 1–3, respectively) with AD that were present only in one of the four investigated cohorts. While successful replication of a discovered association in an independent cohort has become the gold standard in genome-wide association studies for substantiating the real genetic effects, failure to replicate SNP–disease associations does not necessarily indicate that they are false-positive findings. Instead, they might be real genetic contributors that confer population-specific risks due to the genetic heterogeneity of the disease [2, 60, 89, 90]. Other reasons for nonreproducibility can be the lack of statistical power due to insufficient sample sizes, the presence of environmental or gene–gene interactions, and a lack of genotyping information for particular loci in different studies. For instance, small between-population allele frequency differences at an interacting locus may result in a lack of power to detect the main effect of a genuine association signal in independent cohorts [60]. These reasons can also justify why not all previously discovered AD-associated SNPs were replicated in our study.

Of particular interest was to investigate the sex disparity in the genetic basis of AD. Addressing sex differences in biomedical research has been emphasized by the National Institutes of Health as an approach that can eventually bolster the personalized medicine paradigm [14, 15]. Our results revealed a number of new sex-specific genetic contributors to AD at the SNP, gene, and transcriptome levels. For instance, most of the newly detected SNPs, particularly SNPs outside chromosome 19q13, were sex specific as they had significant *p* values either in males or females and, in addition, their odds ratios were significantly different between the two genders. Interestingly, there were two additional subsets of SNPs that were nominally associated with AD in all datasets in one sex while they were nonsignificant in all datasets in the other. Such consistent sex-specific association signals, although weak, might be important in exploring the differences in genetic risk factors of AD between males and females and may demonstrate genome-wide significance in larger samples. Another level of sex disparity was observed in the gene-based and TWA analyses where several genes were significantly associated with AD in either males or females. Also, there were several pathways that were specifically significant in females. These will be further discussed in the following paragraphs.

In the gene-based analysis, *LINC00158*, *MIR155HG*, *MIR155*, *LINC00515*, *MRPL39*, and *JAM2* were significantly associated with AD when the entire sample of individuals and/or only females were analyzed. These genes are located near each other on chromosome 21q21.3 in a ~ 332-kb region. The *APP* gene implicated in early onset familial AD or Down syndrome-related AD [4] is also located 163–449 kb from these genes. There were no AD-associated SNPs with *p* < 5E–08 within their 1 Mb in current or previous studies [43]. However, there were several SNPs with significant *p* values at the suggestive level of associations in that chromosomal region among meta-analysis sets of SNPs under plan 1 (i.e., rs76252969 and rs2298369) and plan 3 (i.e., rs12386284, rs1783012, rs1783013, rs926963, rs1893650, rs2226326, rs2829803, rs2298369, rs2829823, and rs2829832). The SNPs in the 1-Mb upstream/downstream regions of these genes were previously associated with some potential AD risk factors such as type 2 diabetes, hypertension, coronary artery disease, and lipid profile changes at the genome-wide significance level. They have also been associated with traits such as alcohol and nicotine codependence, age at onset of Parkinson’s disease, and pattern recognition memory at the suggestive significance level of association [43]. Furthermore, functional studies have provided insight into the potential roles of some of these genes in AD pathogenesis. For instance, *MIR155HG* and *MIR155* encode two microRNAs. *MIR155* overexpression was previously implicated in downregulation of *complement factor H* (*CFH*) expression in AD and other neurodegenerative diseases which in turn may prevent spontaneous immune system activation [91]. *MRPL39* encodes a mitochondrial ribosomal protein involved in the oxidative–phosphorylation pathway. Impaired mitochondrial function has been reported in neurons of patients with AD [92, 93]. Lunnon et al. [92] reported that the expression levels of *MRPL39* and another nearby gene (i.e., *ATP5J* involved in the oxidative–phosphorylation pathway) were slightly reduced in AD patients compared to controls. *JAM2* encodes a membrane protein found at the tight junctions of epithelial and endothelial cells that acts as an adhesive ligand for immune cells. It belongs to the immunoglobulin superfamily of adhesive molecules that has been implicated in AD pathogenesis [94]. Also, duplication of an ~ 600-kb region on chromosome 21 containing the *JAM2*, *ATP5J*, and *APP* genes has been reported in autosomal dominant AD [95].

In TWA analyses using brain-specific eQTLs data, four probes/genes were associated with AD (two in males and two in females). Also, using eQTLs data from peripheral blood, the expression level of 17 probes/genes passed both the SMR and the HEIDI tests, indicating that variants influencing the expression of these genes may also have pleiotropic effects on developing AD [55, 56]. It should be noted that due to the tissue-specific expression of genes, using data from eQTLs studies on blood is not ideal for capturing associations between the transcriptome levels and AD. However, it increases the power of SMR analysis since such studies take advantage of more samples compared to brain-specific eQTLs studies [55]. Significant SNPs with *p* < 5E–08 were detected within 1 Mb of *MS4A6A* and *UQCC* genes (significant in TWA analyses of blood eQTLs data) in our GWAS or previous reports [43]. SNPs with *p* < 5E–06 were present only in 1-Mb upstream/downstream regions of *ATG10* (significant in brain-specific TWA analyses) in our GWA analyses of females, although several AD-associated SNPs with *p* < 5E–06 were reported in regions around *TRA2A* [64], *IRAK3* [77], and *ESPN* [78]. This is likely indicative of the lack of power of conducted GWAS due to insufficient sample sizes [55].

Taken together, all AD-associated genes in our TWA analyses except *MS4A6A* and *UQCC* can be considered novel potential AD-associated genes. Further functional analyses are needed to explore their potential roles in AD pathogenesis as detected associations do not imply causation. Instead, they provide a list of prioritized candidates for follow-up studies. SNPs in 1-Mb upstream/downstream regions around these genes have been previously associated with some other traits (e.g., autoimmune diseases or serum cholesterol levels) with *p* < 5E–06. Examples include associations of SNPs corresponding to *ABCB9* with college completion and years of education, *ATG10* with vascular dementia, *C9orf72* with amyotrophic lateral sclerosis, frontotemporal lobar degeneration, and response of rheumatoid arthritis patients to anti-TNF treatment, *GNAI3* with total and low-density lipoprotein cholesterol (LDL-C) and major depression, *LPXN* with inflammatory bowel disease, *MED30* with rheumatoid arthritis and fasting blood glucose, *PRDM10* with type 2 diabetes, and *SFN* with high-density lipoprotein cholesterol (HDL-C) [43].

Notably, none of the novel AD-associated genes detected in males were among the significant genes in females and vice versa. Among the significant genes detected in females, a pathologic hexa-nucleotide repeat expansion in the *C9orf72* gene has been linked to frontotemporal dementia and may contribute to AD pathogenesis [96–99]. Also, the *GNAI3* gene was reported to be overexpressed in AD intact mice compared to AD impaired ones [100]. *CRIPAK*, which was among significant genes detected in brain-specific TWA analyses in males, is an inhibitor of the *PAK1* gene [101]. The *PAK* gene family was found to play roles in learning and memory, and the dysregulations were implicated in AD, Huntington disease, and mental retardation [102]. Also, rs1923775 located ~ 700 kb away from *CRIPAK* has shown relatively strong association (*p* = 5.60E–6) with AD in African Americans [88].

Of 26 pathways that were significantly associated with AD in our pathway-based analyses, 12 were not plan specific, seven were specifically significant only under plan 1 (males and females), and seven were specifically significant only in females (i.e., plan 3). Pathways that were significant in more than one plan were mostly involved in processes such as mitochondrial function, lipid metabolism, cell junctions, and immune and inflammatory responses that were implicated in AD [93, 103–106]. There are several lines of evidence in previous empirical studies substantiating the potential roles of some of the detected plan-specific pathways in AD pathogenesis. For instance, it was suggested that deactivation of the epidermal growth factor receptor (EGFR) signaling pathway may attenuate the Aβ-induced memory loss in Drosophila and mice models [107]. Also, the fragmentation and dysfunction of Golgi apparatus, an organelle involved in the posttranslational modifications and trafficking of proteins, has been implicated in AD pathogenesis [108, 109]. The upregulation of the Fas signaling pathway, involved in the apoptosis and modulating immune responses, was reported to contribute to the Aβ-induced cell death and neurodegeneration in AD [110, 111]. Also, dysregulation of the platelet-derived growth factor (PDGF) signaling pathway was suggested to increase Aβ production and contribute to the neurodegeneration in AD [112, 113].

Among the female-specific pathways, G-protein activation is a signal transduction pathway that can modulate the production and action of different intracellular effector proteins. The G protein-coupled receptors play important roles in the initiation and regulation of inflammatory responses such as phagocyte chemotaxis and cytokine production [50, 114]. The pathologically increased inflammatory responses were reported in the brain of patients with AD [93]. Gβγ signaling through the PI3Kγ pathway is involved in the regulation of immune system responses and platelet activation [115]. Also, the ADP signaling, signal amplification, and prostacyclin signaling pathways are involved in the regulation of platelets activation in response to injury or in healthy blood vessels [50]. Platelets, as the major sources of amyloid precursor protein (APP) and Aβ in blood, were reported to be overactivated in AD patients possibly due to their stimulation by injured cerebral endothelial cells or by their cell membrane abnormalities [116, 117]. The glucagon-type ligand receptors are found in the gastrointestinal epithelium and brain neurons. Glucagon-like peptide-1 (GLP-1) has been suggested as a potential treatment to reverse the neurodegeneration in AD and Parkinson’s disease [118, 119].

## Conclusions

In summary, our study revealed significant associations of several SNPs at genome-wide or suggestive levels of significance which were not reported before. Most of the SNPs that were located outside the *APOE* cluster gene region were not in LD with previously discovered AD-associated polymorphisms that had *p* < 5E–06 (Table 2). These SNPs were mapped to 21 genes in 19 chromosomal regions. Of these, 8 genes/regions had not been associated with AD in previous GWAS with *p* < 5E–06. Also, 26 genes located outside the chromosome 19q13 region, and 26 pathways, showed evidence of associations with AD at the FDR level of 0.05 in our TWA, gene-based, and pathway-based analyses. Thirteen of these 26 genes were located in chromosomal regions with no AD-associated SNPs at the genome-wide or suggestive level of significance. Most of the significantly detected SNPs and genes as well as several AD-associated pathways were sex specific, indicating sex disparities in the genetic basis of AD. By detecting a number of novel potential AD-associated SNPs and discovering suggestive associations of several genes and transcripts, our study provides new insight into the genetic architecture of AD. Particularly, identifying sex-specific genetic contributors can advance our understanding of AD pathogenesis.

Despite the rigor of this study, there are some limitations. The case/control status in the four cohorts used in this study was mainly determined clinically. The routine clinical diagnosis of AD based on the symptoms and neurologic examinations may not provide the optimal case/control classification. Instead, the National Institute on Aging and the Alzheimer’s Association suggested that integrating additional paraclinical tests (e.g., histopathologic findings in brain biopsy, measuring AD-related cerebrospinal fluid (CSF) biomarkers, or detecting neurodegeneration by the imaging study) into the diagnostic protocols can aid researchers to more accurately identify AD patients and healthy controls [120, 121]. Beach et al. [122] investigated the accuracy of clinical diagnosis of AD by comparing such diagnoses to the histopathology findings from brain autopsies in a sample of 1198 subjects. They found that the sensitivity and specificity of clinical diagnostic classification were 70.9–87.3% and 44.3–70.8%, respectively, indicating a relatively high possibility of clinically false-negative and false-positive classification of subjects as controls and cases, respectively [122]. Finally, since the power of GWA analyses is affected by the sample sizes, and in particular the number of cases, the current study with 2741 cases and 14,739 controls may not have the optimal power. Further studies, possibly with larger sample sizes, are needed to clarify the genotype–phenotype relationships in AD.

## Additional files

Additional file 1: **Table S1.** Cases and controls included. **Table S2.** QC-passed SNPs analyzed in datasets. **Table S3.** Genomic inflation factors (*λ* values) from logistic regression models. **Table S4–S6.** Replicated set of SNPs detected under analysis plan 1 (males and females), plan 2 (only males), and plan 3 (only females). **Table S7–S9.** Nonreplicated set of SNPs detected under analysis plan 1 (males and females), plan 2 (only males), and plan 3 (only females). **Table S10–S12.** Meta-analysis set of SNPs detected under analysis plan 1 (males and females), plan 2 (only males), and plan 3 (only females). **Table 13.** LD information about newly detected SNPs under plans 1–3 for which proxy AD-associated loci exist in 1-Mb flanking regions [8, 9]. **Table S14.** Coding schema used to determine *APOE* genotypes. **Table 15.** Information about LD between *APOE* SNPs and AD-associated SNPs located on chromosome 19 [8]. **Table S16–S17.** Wald χ^2^ test to compare ORs of SNPs between males and females for SNPs that were specifically significant in males and in females. **Figure S1–S6.** Manhattan plot and QQ plot of genome-wide association results under analysis plan 1 (males and females), plan 2 (only males), and plan 3 (only females). **Supporting Acknowledgment.** Furthre information about the four cohorts under consideration. (DOCX 284 kb)

Additional file 2: Detailed information about the AD-associated SNPs under analysis plan 1 (males and females). (XLSX 72 kb)

Additional file 3: Detailed information about the AD-associated SNPs under analysis plan 2 (only males). (XLSX 57 kb)

Additional file 4: Detailed information about the AD-associated SNPs under analysis plan 3 (only females). (XLSX 72 kb)

Additional file 5: Detailed information about the nominally AD-associated SNPs with the opposite pattern of significance in males and females. (XLSX 40 kb)

Additional file 6: Detailed information about the results from the Wald χ2 test to compare ORs of SNPs between males and females. (XLSX 107 kb)

## Abbreviations

AD: Alzheimer’s disease
APOE: Apolipoprotein E
APP: Amyloid precursor protein
Aβ: Amyloid beta
BA24: Brodmann Area 24
BA9: Brodmann Area 9
CEU: Utah Residents with Northern and Western European Ancestry
CHS: Cardiovascular Health Study
dbGaP: Database of Genotypes and Phenotypes
eQTLs: Expression quantitative trait loci
EUR: European population haplotypes
fastBAT: Fast Set-Based Association Test
FDR: False discovery rate
FHS: Framingham Heart Study
GCTA: Genome-wide Complex Trait Analysis
GLMM: Generalized linear mixed model
GLP-1: Glucagon-like peptide-1
GRASP: Genome-wide Repository of Associations between SNPs and Phenotypes
GSEA: Gene set enrichment analysis
GWA: Genome-wide association analysis
GWAMA: Genome-wide association meta-analysis
GWAS: Genome-wide association study
HDL-C: High-density lipoprotein cholesterol
HEIDI: Heterogeneity in Dependent Instruments
hg19: Human Genome Build 19
hg38: Human Genome Build 38
HRS: Health and Retirement Study
IBD: Identity-by-descent
ICD-9: International Classification of Disease Codes, ninth revision
ID: Identifier
IRB: Institutional review board
KING: Kinship-Based Inference for GAWS
LD: Linkage disequilibrium
LDL-C: Low-density lipoprotein cholesterol
MSigDB: Molecular Signatures Database
NCBI: National Center for Biotechnology Information
NIA-LOADFS: Late-Onset Alzheimer’s Disease Family Study from the National Institute on Aging
OR: Odds ratio
PC: Principal component
PCA: Principal component analysis
PID: Pathway Interaction Database
QC: Quality control
SHAPEIT: Segmented Haplotype Estimation and Imputation Tool
SHARe: Framingham SNP Health Association Resource
SMR: Summary-data-based Mendelian Randomization
SNP: Single-nucleotide polymorphism
STAMPEED: SNP Typing for Association with Multiple Phenotypes from Existing Epidemiologic Data
TWA: Transcriptome-wide association analysis
UCSC: University of California, Santa Cruz
