# Supplementary material for: Genome-wide analysis of genetic predisposition to Alzheimer’s disease and related sex disparities
Source: Alzheimers Res Ther. 2019 Jan 12;11:5. doi: 10.1186/s13195-018-0458-8 (PMC6330399; doi:10.1186/s13195-018-0458-8)
Supplement: Supplementary file 1 — Table S1. Cases and controls included. Table S2. QC-passed SNPs analyzed in datasets. Table S3. Genomic inflation factors (λ values) from logistic regression models. Table S4–S6. Replicated set of SNPs detected under analysis plan 1 (males and females), plan 2 (only males), and plan 3 (only females). Table S7–S9. Nonreplicated set of SNPs detected under analysis plan 1 (males and females), plan 2 (only males), and plan 3 (only females). Table S10–S12. Meta-analysis set of SNPs detected under analysis plan 1 (males and females), plan 2 (only males), and plan 3 (only females). Table 13. LD information about newly detected SNPs under plans 1–3 for which proxy AD-associated loci exist in 1-Mb flanking regions [8, 9]. Table S14. Coding schema used to determine APOE genotypes. Table 15. Information about LD between APOE SNPs and AD-associated SNPs located on chromosome 19 [8]. Table S16–S17. Wald χ2 test to compare ORs of SNPs between males and females for SNPs that were specifically significant in males and in females. Figure S1–S6. Manhattan plot and QQ plot of genome-wide association results under analysis plan 1 (males and females), plan 2 (only males), and plan 3 (only females). Supporting Acknowledgment. Furthre information about the four cohorts under consideration. (DOCX 284 kb) [file 13195_2018_458_MOESM1_ESM.docx]

**Genome-wide analysis of genetic predisposition to Alzheimer’s disease and related sex disparities**

Alireza Nazarian^*^, Anatoliy I. Yashin, and Alexander M. Kulminski^*^

Biodemography of Aging Research Unit, Social Science Research Institute, Duke University, Durham, NC, USA

*Corresponding Authors:

Alireza Nazarian and Alexander M. Kulminski

Duke University

Bio-demography of Aging Research Unit

Social Science Research Institute

Erwin Mill Building, 2024 W. Main St.

Durham, NC 27705

Emails: [alireza.nazarian@duke.edu](mailto:alireza.nazarian@duke.edu) and [kulminsk@duke.edu](mailto:kulminsk@duke.edu)

**Additional File 1**

**Table S1: Numbers of cases and controls included in this study**

| **Study** | **Plan 1** | | | **Plan 2** | | | **Plan 3** | | |
| --- | --- | --- | --- | --- | --- | --- | --- | --- | --- |
|  | **Case** | **Control** | **Total** | **Case** | **Control** | **Total** | **Case** | **Control** | **Total** |
| **LOADFS** | 1850 | 1866 | 3716 | 644 | 752 | 1396 | 1206 | 1114 | 2320 |
| **FHS** | 413 | 3996 | 4409 | 138 | 1856 | 1994 | 275 | 2140 | 2415 |
| **CHS** | 197 | 3000 | 3197 | 71 | 1199 | 1270 | 126 | 1801 | 1927 |
| **HRS** | 281 | 5877 | 6158 | 99 | 2530 | 2629 | 182 | 3347 | 3529 |
| **Total** | 2741 | 14739 | 17480 | 952 | 6337 | 7289 | 1789 | 8402 | 10191 |

Plan 1: males and females; Plan 2: only males; Plan 3: only females

LOADFS: Late-Onset Alzheimer's Disease Family Study from the National Institute on Aging [1]; FHS: Framingham Heart Study [2–4]; CHS: Cardiovascular Health Study [5]; HRS: the University of Michigan Health and Retirement Study [6]

**Table S2: Numbers of QC-passed SNPs that were analyzed in datasets under consideration**

| **Study** | **Plan 1** | | | **Plan 2** | | | **Plan 3** | | |
| --- | --- | --- | --- | --- | --- | --- | --- | --- | --- |
|  | **Genotyped** | **Imputed** | **Total** | **Genotyped** | **Imputed** | **Total** | **Genotyped** | **Imputed** | **Total** |
| **LOADFS** | 554561 | 1277493 | 1832054 | 554383 | 1276804 | 1831187 | 554500 | 1277237 | 1831737 |
| **FHS** | 400314 | 1161099 | 1561413 | 399665 | 1159717 | 1559382 | 400107 | 1160938 | 1561045 |
| **CHS** | 324696 | 1373264 | 1697960 | 324491 | 1365341 | 1689832 | 324662 | 1371799 | 1696461 |
| **HRS** | 1552941 | 440946 | 1993887 | 1546419 | 439943 | 1986362 | 1552278 | 440797 | 1993075 |

Abbreviations: QC = quality control; SNP = single-nucleotide polymorphism

Plan 1: males and females; Plan 2: only males; Plan 3: only females

LOADFS: Late-Onset Alzheimer's Disease Family Study from the National Institute on Aging [1]; FHS: Framingham Heart Study [2–4]; CHS: Cardiovascular Health Study [5]; HRS: the University of Michigan Health and Retirement Study [6]

**Table S3: Genomic inflation factors (λ values) from logistic regression models fitted by PLINK package** [7]

| **Study** | **Plan 1** | **Plan 2** | **Plan 3** |
| --- | --- | --- | --- |
| **LOADFS** | 1.08 | 1.07 | 1.03 |
| **FHS** | 1.09 | 1.08 | 1.06 |
| **CHS** | ~1.00 | ~1.00 | ~1.00 |
| **HRS** | 1.01 | 1.01 | 1.02 |

Plan 1: males and females; Plan 2: only males; Plan 3: only females

LOADFS: Late-Onset Alzheimer's Disease Family Study from the National Institute on Aging [1]; FHS: Framingham Heart Study [2–4]; CHS: Cardiovascular Health Study [5]; HRS: the University of Michigan Health and Retirement Study [6]

**Table S4: List of replicated set of SNPs detected under analysis Plan 1 (males and females)**

| **Previously Detected** | | | | | **Newly Detected** | | | |
| --- | --- | --- | --- | --- | --- | --- | --- | --- |
| **Genome-wide Level** | | | **Suggestive Level** | | **Genome-wide Level** | | **Suggestive Level** | |
| **Chr19** | | **Others** | **Chr19** | **Others** | **Chr19** | **Others** | **Chr19** | **Others** |
| rs10405693 | rs7259620 |  | rs440277 |  | rs58446550 |  | rs57537848 | rs62402815 |
| rs10402271 | rs405509 |  |  |  | rs73048293 |  |  |  |
| rs6859 | rs769449 |  |  |  | rs56317818 |  |  |  |
| rs71352238 | rs769450 |  |  |  | rs12462573 |  |  |  |
| rs157580 | rs429358 |  |  |  | rs11668861 |  |  |  |
| rs2075650 | rs7412 |  |  |  | rs3852860 |  |  |  |
| rs157582 | rs56131196 |  |  |  | rs283815 |  |  |  |
| rs8106922 | rs4420638 |  |  |  | rs34095326 |  |  |  |
| rs1160985 |  |  |  |  | rs75627662 |  |  |  |
| rs405697 |  |  |  |  | rs483082 |  |  |  |

Single-nucleotide polymorphisms (SNPs) in green were specifically significant under Plan 1 (i.e., they were not among replicated, non-replicated, or meta-analysis sets of SNPs under the other two plans).

Genome-wide and suggestive significance levels of associations refer to P < 5E-08 and 5E-08 ≤ P < 5E-06, respectively.

**Table S5: List of replicated set of SNPs detected under analysis Plan 2 (only males)**

| **Previously Detected** | | | | **Newly Detected** | | | |
| --- | --- | --- | --- | --- | --- | --- | --- |
| **Genome-wide Level** | | **Suggestive Level** | | **Genome-wide Level** | | **Suggestive Level** | |
| **Chr19** | **Others** | **Chr19** | **Others** | **Chr19** | **Others** | **Chr19** | **Others** |
| rs71352238 |  | rs6859 | rs9882471 | rs283815 |  | rs56317818 | rs9918162 |
| rs2075650 |  | rs1160985 |  | rs34095326 |  | rs12462573 | rs726411 |
| rs157582 |  | rs7259620 |  | rs75627662 |  |  |  |
| rs769449 |  | rs405509 |  | rs483082 |  |  |  |
| rs429358 |  |  |  |  |  |  |  |
| rs56131196 |  |  |  |  |  |  |  |
| rs4420638 |  |  |  |  |  |  |  |

Single-nucleotide polymorphisms (SNPs) in blue were specifically significant under Plan 2 (i.e., they were not among replicated, non-replicated, or meta-analysis sets of SNPs under the other two plans).

Genome-wide and suggestive significance levels of associations refer to P < 5E-08 and 5E-08 ≤ P < 5E-06, respectively.

**Table S6: List of replicated set of SNPs detected under analysis Plan 3 (only females)**

| **Previously Detected** | | | | | **Newly Detected** | | | |
| --- | --- | --- | --- | --- | --- | --- | --- | --- |
| **Genome-wide Level** | | | **Suggestive Level** | | **Genome-wide Level** | | **Suggestive Level** | |
| **Chr19** | | **Others** | **Chr19** | **Others** | **Chr19** | **Others** | **Chr19** | **Others** |
| rs6859 | rs769449 |  | rs10405693 |  | rs283815 | rs62402815 | rs58446550 |  |
| rs71352238 | rs429358 |  | rs10402271 |  | rs34095326 |  | rs56317818 |  |
| rs157580 | rs439401 |  |  |  | rs75627662 |  | rs12462573 |  |
| rs2075650 | rs584007 |  |  |  | rs483082 |  |  |  |
| rs157582 | rs56131196 |  |  |  |  |  |  |  |
| rs1160985 | rs4420638 |  |  |  |  |  |  |  |
| rs7259620 |  |  |  |  |  |  |  |  |

Single-nucleotide polymorphisms (SNPs) in red were specifically significant under Plan 3 (i.e., they were not among replicated, non-replicated, or meta-analysis sets of SNPs under the other two plans).

Genome-wide and suggestive significance levels of associations refer to P < 5E-08 and 5E-08 ≤ P < 5E-06, respectively.

**Table S7: List of non-replicated set of SNPs detected under analysis Plan 1 (males and females)**

| **Previously Detected** | | | | **Newly Detected** | | | | | |
| --- | --- | --- | --- | --- | --- | --- | --- | --- | --- |
| **Genome-wide Level** | | **Suggestive Level** | | **Genome-wide Level** | | **Suggestive Level** | | | |
| **Chr19** | **Others** | **Chr19** | **Others** | **Chr19** | **Others** | **Chr19** | **Others** | | |
| rs584007 |  | rs2965164 | rs7591606 | rs28399654 |  | rs12459810 | rs116593898 | rs217309 | rs850946 |
| rs439401 |  | rs2965109 | rs11038106 |  |  | rs406456 | rs77657034 | rs55942513 | rs1948063 |
|  |  | rs2927439 | rs9597722 |  |  |  | rs16830960 | rs55941818 | rs1572067 |
|  |  | rs2965169 | rs723804 |  |  |  | rs2243133 | rs4332034 | rs17629743 |
|  |  | rs283814 | rs17697225 |  |  |  | rs75430555 | rs10248805 | rs7175042 |
|  |  |  | rs2065706 |  |  |  | rs4146582 | rs17730621 | rs16962705 |
|  |  |  |  |  |  |  | rs1516700 | rs2237355 | rs12910448 |
|  |  |  |  |  |  |  | rs17018613 | rs2551790 | rs2233083 |
|  |  |  |  |  |  |  | rs6848489 | rs116517362 | rs12450778 |
|  |  |  |  |  |  |  | rs1834622 | rs12766562 | rs7221498 |
|  |  |  |  |  |  |  | rs16874911 | rs10902230 | rs17081147 |
|  |  |  |  |  |  |  | rs2101881 | rs10794341 | rs111436800 |
|  |  |  |  |  |  |  | rs73471670 | rs11038108 |  |

Single-nucleotide polymorphisms (SNPs) in green were specifically significant under Plan 1 (i.e., they were not among replicated, non-replicated, or meta-analysis sets of SNPs under the other two plans).

Genome-wide and suggestive significance levels of associations refer to P < 5E-08 and 5E-08 ≤ P < 5E-06, respectively.

**Table S8: List of non-replicated set of SNPs detected under analysis Plan 2 (only males)**

| **Previously Detected** | | | | **Newly Detected** | | | | | |
| --- | --- | --- | --- | --- | --- | --- | --- | --- | --- |
| **Genome-wide Level** | | **Suggestive Level** | | **Genome-wide Level** | | **Suggestive Level** | | | |
| **Chr19** | **Others** | **Chr19** | **Others** | **Chr19** | **Others** | **Chr19** | **Others** | | |
| rs157580 |  | rs8106922 | rs4679840 |  |  | rs2302525 | rs6413942 | rs28384174 | rs2904502 |
| rs439401 |  | rs769450 |  |  |  |  | rs4471236 | rs6838042 | rs79722180 |
| rs584007 |  | rs7412 |  |  |  |  | rs4656009 | rs7686186 | rs2041769 |
|  |  |  |  |  |  |  | rs10737721 | rs6852613 | rs2930120 |
|  |  |  |  |  |  |  | rs17754357 | rs77104831 | rs2160243 |
|  |  |  |  |  |  |  | rs116799779 | rs75462348 | rs2160244 |
|  |  |  |  |  |  |  | rs13016495 | rs10795162 | rs4942587 |
|  |  |  |  |  |  |  | rs11127861 | rs7071303 | rs62037093 |
|  |  |  |  |  |  |  | rs34925435 | rs11044882 | rs12910448 |
|  |  |  |  |  |  |  | rs28385458 | rs10878688 | rs73165159 |
|  |  |  |  |  |  |  | rs7616299 | rs741290 |  |

Single-nucleotide polymorphisms (SNPs) in blue were specifically significant under Plan 2 (i.e., they were not among replicated, non-replicated, or meta-analysis sets of SNPs under the other two plans).

Genome-wide and suggestive significance levels of associations refer to P < 5E-08 and 5E-08 ≤ P < 5E-06, respectively.

**Table S9: List of non-replicated set of SNPs detected under analysis Plan 3 (only females)**

| **Previously Detected** | | | | **Newly Detected** | | | | | |
| --- | --- | --- | --- | --- | --- | --- | --- | --- | --- |
| **Genome-wide Level** | | **Suggestive Level** | | **Genome-wide Level** | | **Suggestive Level** | | | |
| **Chr19** | **Others** | **Chr19** | **Others** | **Chr19** | **Others** | **Chr19** | **Others** | | |
| rs8106922 |  |  | rs1359176 | rs3852860 |  | rs12609754 | rs11118774 | rs62433246 | rs10794341 |
| rs405697 |  |  |  |  |  | rs11668861 | rs11678276 | rs1105056 | rs1938923 |
| rs405509 |  |  |  |  |  | rs35879138 | rs34779859 | rs7779578 | rs850946 |
| rs769450 |  |  |  |  |  |  | rs57985280 | rs1062851 | rs1948063 |
| rs7412 |  |  |  |  |  |  | rs2333450 | rs62510850 | rs10876280 |
|  |  |  |  |  |  |  | rs72708337 | rs7000333 | rs6572843 |
|  |  |  |  |  |  |  | rs67220167 | rs73327271 | rs325403 |
|  |  |  |  |  |  |  | rs2255273 | rs13292007 | rs62043827 |
|  |  |  |  |  |  |  | rs78552253 | rs41306812 | rs10514426 |
|  |  |  |  |  |  |  | rs41271129 | rs17484254 | rs12450778 |
|  |  |  |  |  |  |  | rs62405605 | rs1538431 | rs6065093 |
|  |  |  |  |  |  |  | rs41500045 | rs10902230 | rs6071592 |

Single-nucleotide polymorphisms (SNPs) in red were specifically significant under Plan 3 (i.e., they were not among replicated, non-replicated, or meta-analysis sets of SNPs under the other two plans).

Genome-wide and suggestive significance levels of associations refer to P < 5E-08 and 5E-08 ≤ P < 5E-06, respectively.

**Table S10: List of meta-analysis set of SNPs detected under analysis Plan 1 (males and females)**

| **Previously Detected** | | | | **Newly Detected** | | | |
| --- | --- | --- | --- | --- | --- | --- | --- |
| **Genome-wide Level** | | **Suggestive Level** | | **Genome-wide Level** | | **Suggestive Level** | |
| **Chr19** | **Others** | **Chr19** | **Others** | **Chr19** | **Others** | **Chr19** | **Others** |
|  |  | rs8103315 |  | rs76366838 |  | rs35879138 | rs10953322 |
|  |  | rs10426423 |  | rs115881343 |  |  | rs4961664 |
|  |  | rs1871047 |  |  |  |  | rs2282079 |
|  |  |  |  |  |  |  | rs10794342 |
|  |  |  |  |  |  |  | rs9555561 |
|  |  |  |  |  |  |  | rs912322 |
|  |  |  |  |  |  |  | rs8070114 |
|  |  |  |  |  |  |  | rs1497197 |
|  |  |  |  |  |  |  | rs35242772 |
|  |  |  |  |  |  |  | rs76252969 |
|  |  |  |  |  |  |  | rs2298369 |

Single-nucleotide polymorphisms (SNPs) in green were specifically significant under Plan 1 (i.e., they were not among replicated, non-replicated, or meta-analysis sets of SNPs under the other two plans).

Genome-wide and suggestive significance levels of associations refer to P < 5E-08 and 5E-08 ≤ P < 5E-06, respectively.

**Table S11: List of meta-analysis set of SNPs detected under analysis Plan 2 (only males)**

| **Previously Detected** | | | | **Newly Detected** | | | |
| --- | --- | --- | --- | --- | --- | --- | --- |
| **Genome-wide Level** | | **Suggestive Level** | | **Genome-wide Level** | | **Suggestive Level** | |
| **Chr19** | **Others** | **Chr19** | **Others** | **Chr19** | **Others** | **Chr19** | **Others** |
|  |  | rs8103315 |  |  |  | rs3852860 | rs9862849 |
|  |  |  |  |  |  |  | rs5969117 |

Single-nucleotide polymorphisms (SNPs) in blue were specifically significant under Plan 2 (i.e., they were not among replicated, non-replicated, or meta-analysis sets of SNPs under the other two plans).

Genome-wide and suggestive significance levels of associations refer to P < 5E-08 and 5E-08 ≤ P < 5E-06, respectively.

**Table S12: List of meta-analysis set of SNPs detected under analysis Plan 3 (only females)**

| **Previously Detected** | | | | **Newly Detected** | | | | | |
| --- | --- | --- | --- | --- | --- | --- | --- | --- | --- |
| **Genome-wide Level** | | **Suggestive Level** | | **Genome-wide Level** | | **Suggestive Level** | | | |
| **Chr19** | **Others** | **Chr19** | **Others** | **Chr19** | **Others** | **Chr19** | **Others** | | |
|  |  |  |  | rs73048293 |  | rs115881343 | rs7561207 | rs1172922 | rs1893650 |
|  |  |  |  | rs57537848 |  |  | rs17675640 | rs73156187 | rs2226326 |
|  |  |  |  | rs76366838 |  |  | rs6838792 | rs7963314 | rs2829803 |
|  |  |  |  |  |  |  | rs895681 | rs12386284 | rs2298369 |
|  |  |  |  |  |  |  | rs11969759 | rs1783012 | rs2829823 |
|  |  |  |  |  |  |  | rs10947230 | rs1783013 | rs2829832 |
|  |  |  |  |  |  |  | rs7774197 | rs926963 |  |

Single-nucleotide polymorphisms (SNPs) in red were specifically significant under Plan 3 (i.e., they were not among replicated, non-replicated, or meta-analysis sets of SNPs under the other two plans).

Genome-wide and suggestive significance levels of associations refer to P < 5E-08 and 5E-08 ≤ P < 5E-06, respectively.

**Table S13: LD information about newly detected SNPs under Plans 1-3 for which proxy AD-associated loci exist in their 1 Mb flanking regions** [8,9]

| **Chromosome** | **SNP** | **Position** | **Proxy** | **Proxy-Position** | **Proxy-P-value** | **Proxy-Gene** | **Reference** | **D'** | **R^2^** | **Chi-square** | **P-value** |
| --- | --- | --- | --- | --- | --- | --- | --- | --- | --- | --- | --- |
| 2 | rs34779859 | 127135192 | rs744373 | 127137039 | **2.6E-14** | *BIN1,NIFKP9* | [10] | 0.510 | 0.211 | 41.711 | **<0.0001** |
| 11 | rs10794342 | 924904 | rs10751667 | 941941 | **6.00E-07** | *AP2A2* | [11] | 1.000 | 0.698 | 138.152 | **<0.0001** |
| 19 | rs12459810 | 44746404 | rs8103315 | 44750911 | **1.9E-21** | *BCL3* | [12] | 1.000 | 0.454 | 89.901 | **<0.0001** |
| 19 | rs58446550 | 44825123 | rs4803763 | 44854034 | **9.9E-69** | *PVRL2* | [12] | 0.920 | 0.603 | 119.370 | **<0.0001** |
| 19 | rs73048293 | 44837479 | rs4803763 | 44854034 | **9.9E-69** | *PVRL2* | [12] | 0.924 | 0.709 | 140.466 | **<0.0001** |
| 19 | rs57537848 | 44850787 | rs4803763 | 44854034 | **9.9E-69** | *PVRL2* | [12] | 1.000 | 0.409 | 81.023 | **<0.0001** |
| 19 | rs56317818 | 44856329 | rs4803763 | 44854034 | **9.9E-69** | *PVRL2* | [12] | 1.000 | 0.953 | 188.666 | **<0.0001** |
| 19 | rs12462573 | 44856449 | rs4803763 | 44854034 | **9.9E-69** | *PVRL2* | [12] | 1.000 | 0.953 | 188.666 | **<0.0001** |
| 19 | rs11668861 | 44877713 | rs6857 | 44888997 | **2.18e-320** | *PVRL2* | [12] | 0.777 | 0.126 | 24.926 | **<0.0001** |
| 19 | rs406456 | 44879460 | rs6857 | 44888997 | **2.18e-320** | *PVRL2* | [12] | 0.555 | 0.102 | 20.277 | **<0.0001** |
| 19 | rs3852860 | 44879709 | rs6859 | 44878777 | **5.6E-74** | *PVRL2* | [12] | 1.000 | 0.554 | 109.607 | **<0.0001** |
| 19 | rs35879138 | 44879882 | rs519113 | 44873027 | **5.1E-39** | *PVRL2* | [13] | 0.940 | 0.355 | 70.320 | **<0.0001** |
| 19 | rs283815 | 44887076 | rs6857 | 44888997 | **2.18e-320** | *PVRL2* | [12] | 1.000 | 0.788 | 156.031 | **<0.0001** |
| 19 | rs34095326 | 44892587 | rs6857 | 44888997 | **2.18e-320** | *PVRL2* | [12] | 1.000 | 0.589 | 116.652 | **<0.0001** |
| 19 | rs76366838 | 44896639 | rs6857 | 44888997 | **2.18e-320** | *PVRL2* | [12] | 0.822 | 0.101 | 19.995 | **<0.0001** |
| 19 | rs115881343 | 44899959 | rs6857 | 44888997 | **2.18e-320** | *PVRL2* | [12] | 1.000 | 0.127 | 25.226 | **<0.0001** |
| 19 | rs75627662 | 44910319 | rs6857 | 44888997 | **2.18e-320** | *PVRL2* | [12] | 0.580 | 0.316 | 62.479 | **<0.0001** |
| 19 | rs483082 | 44912921 | rs6857 | 44888997 | **2.18e-320** | *PVRL2* | [12] | 0.765 | 0.461 | 91.229 | **<0.0001** |

Abbreviations: AD = Alzheimer’s disease; LD = linkage disequilibrium; SNP = single-nucleotide polymorphism

**Table S14. Coding schema used to determine *APOE* genotypes**

| ***APOE* SNPs** | **rs7412_CC** | **rs7412_CT** | **rs7412_TT** |
| --- | --- | --- | --- |
| **rs429358_TT** | ε3ε3 | ε2ε3 | ε2ε2 |
| **rs429358_TC** | ε3ε4 | ε2ε4 | -- |
| **rs429358_CC** | ε4ε4 | -- | -- |

Abbreviations: *APOE* = *Apolipoprotein E*

**Table S15: Information about LD between *APOE* SNPs and AD-associated SNPs located on chromosome 19** [8]

| **SNP** | **rs429358** | | | | **rs7412** | | | |
| --- | --- | --- | --- | --- | --- | --- | --- | --- |
|  | **D'** | **R^2^** | **Chi-square** | **p-value** | **D'** | **R^2^** | **Chi-square** | **P-value** |
| **Newly Detected SNPs** | | | | | | | | |
| rs58446550 | 0.356 | 0.045 | 8.842 | 0.003 | 0.797 | 0.027 | 5.388 | 0.020 |
| rs73048293 | 0.304 | 0.038 | 7.499 | 0.006 | 0.776 | 0.022 | 4.383 | 0.036 |
| rs57537848 | 0.352 | 0.025 | 4.952 | 0.026 | 0.365 | 0.009 | 1.748 | 0.186 |
| rs56317818 | 0.273 | 0.039 | 7.629 | 0.006 | 0.737 | 0.016 | 3.134 | 0.077 |
| rs12462573 | 0.273 | 0.039 | 7.629 | 0.006 | 0.737 | 0.016 | 3.134 | 0.077 |
| rs11668861 | 0.627 | 0.072 | 14.215 | 0.000 | 1.000 | 0.060 | 11.833 | 0.001 |
| rs3852860 | 0.646 | 0.061 | 12.045 | 0.001 | 0.226 | 0.005 | 1.044 | 0.307 |
| rs283815 | 0.925 | 0.590 | 116.889 | **<0.0001** | 0.193 | 0.008 | 1.666 | 0.197 |
| rs34095326 | 0.709 | 0.338 | 66.883 | **<0.0001** | 1.000 | 0.010 | 2.011 | 0.156 |
| rs75627662 | 0.748 | 0.460 | 91.033 | **<0.0001** | 1.000 | 0.269 | 53.279 | **<0.0001** |
| rs483082 | 0.963 | 0.639 | 126.549 | **<0.0001** | 1.000 | 0.226 | 44.701 | **<0.0001** |
| rs12459810 | 0.086 | 0.003 | 0.603 | 0.437 | 0.552 | 0.011 | 2.218 | 0.136 |
| rs28399654 | 1.000 | 0.006 | 1.101 | 0.294 | 0.358 | 0.047 | 9.347 | 0.002 |
| rs406456 | 0.405 | 0.048 | 9.441 | 0.002 | 0.275 | 0.004 | 0.774 | 0.379 |
| rs35879138 | 0.227 | 0.030 | 5.939 | 0.015 | 0.048 | 0.001 | 0.257 | 0.612 |
| rs76366838 | 0.827 | 0.117 | 23.084 | **<0.0001** | 1.000 | 0.003 | 0.510 | 0.475 |
| rs115881343 | 1.000 | 0.146 | 28.816 | **<0.0001** | 1.000 | 0.002 | 0.435 | 0.510 |
| rs2604880 | 0.197 | 0.025 | 4.899 | 0.027 | 0.391 | 0.004 | 0.718 | 0.397 |
| rs2302525 | 1.000 | 0.006 | 1.101 | 0.294 | 0.144 | 0.008 | 1.509 | 0.219 |
| rs12609754 | 0.200 | 0.010 | 1.949 | 0.163 | 0.338 | 0.007 | 1.378 | 0.241 |
| rs78807487 | 0.099 | 0.008 | 1.602 | 0.206 | 0.257 | 0.001 | 0.240 | 0.624 |
| rs11672827 | 0.051 | 0.002 | 0.334 | 0.563 | 0.378 | 0.003 | 0.655 | 0.418 |
| **Previously Detected SNPs** | | | | | | | | |
| rs2965164 | 0.122 | 0.001 | 0.263 | 0.608 | 0.021 | 0.000 | 0.015 | 0.904 |
| rs2965109 | 0.081 | 0.001 | 0.188 | 0.665 | 0.097 | 0.001 | 0.191 | 0.662 |
| rs2927439 | 0.217 | 0.005 | 0.976 | 0.323 | 0.313 | 0.014 | 2.787 | 0.095 |
| rs2965169 | 0.136 | 0.002 | 0.448 | 0.504 | 0.275 | 0.009 | 1.838 | 0.175 |
| rs8103315 | 0.107 | 0.001 | 0.115 | 0.734 | 0.599 | 0.006 | 1.186 | 0.276 |
| rs10405693 | 0.356 | 0.045 | 8.842 | 0.003 | 0.797 | 0.027 | 5.388 | 0.020 |
| rs10402271 | 0.356 | 0.045 | 8.842 | 0.003 | 0.797 | 0.027 | 5.388 | 0.020 |
| rs10426423 | 0.192 | 0.004 | 0.856 | 0.355 | 0.130 | 0.001 | 0.128 | 0.721 |
| rs1871047 | 0.192 | 0.004 | 0.856 | 0.355 | 0.130 | 0.001 | 0.128 | 0.721 |
| rs440277 | 0.641 | 0.041 | 8.147 | 0.004 | 0.033 | 0.000 | 0.007 | 0.933 |
| rs6859 | 0.585 | 0.090 | 17.807 | <0.0001 | 0.658 | 0.025 | 4.915 | 0.027 |
| rs283814 | 1.000 | 0.015 | 2.988 | 0.084 | 1.000 | 0.005 | 0.978 | 0.323 |
| rs71352238 | 0.750 | 0.543 | 107.507 | **<0.0001** | 1.000 | 0.015 | 2.885 | 0.089 |
| rs157580 | 0.924 | 0.109 | 21.641 | **<0.0001** | 0.386 | 0.018 | 3.471 | 0.062 |
| rs2075650 | 0.779 | 0.565 | 111.956 | **<0.0001** | 1.000 | 0.014 | 2.783 | 0.095 |
| rs157582 | 0.925 | 0.590 | 116.889 | **<0.0001** | 0.193 | 0.008 | 1.666 | 0.197 |
| rs8106922 | 1.000 | 0.134 | 26.485 | **<0.0001** | 1.000 | 0.044 | 8.667 | 0.003 |
| rs1160985 | 1.000 | 0.155 | 30.685 | **<0.0001** | 0.083 | 0.000 | 0.068 | 0.794 |
| rs405697 | 0.880 | 0.052 | 10.239 | 0.001 | 1.000 | 0.022 | 4.331 | 0.037 |
| rs7259620 | 1.000 | 0.155 | 30.685 | **<0.0001** | 0.083 | 0.000 | 0.068 | 0.794 |
| rs405509 | 0.650 | 0.087 | 17.255 | <0.0001 | 1.000 | 0.073 | 14.487 | 0.000 |
| rs769449 | 1.000 | 0.799 | 158.233 | **<0.0001** | 1.000 | 0.012 | 2.388 | 0.122 |
| rs769450 | 1.000 | 0.134 | 26.485 | **<0.0001** | 1.000 | 0.044 | 8.667 | 0.003 |
| rs439401 | 1.000 | 0.123 | 24.295 | **<0.0001** | 1.000 | 0.040 | 7.951 | 0.005 |
| rs584007 | 1.000 | 0.123 | 24.295 | **<0.0001** | 1.000 | 0.040 | 7.951 | 0.005 |
| rs56131196 | 0.964 | 0.719 | 142.270 | **<0.0001** | 1.000 | 0.020 | 3.860 | 0.050 |
| rs4420638 | 0.964 | 0.719 | 142.270 | **<0.0001** | 1.000 | 0.020 | 3.860 | 0.050 |

Abbreviations: AD = Alzheimer’s disease; *APOE* = *Apolipoprotein E*; LD = linkage disequilibrium; SNP = single-nucleotide polymorphism

Bold p-values indicate the SNP of interest was in LD with APOE SNP(s).

**Table S16:** **Wald chi-square test to compare odds ratios of single-nucleotide polymorphisms (SNPs) between males and females for SNPs that were specifically significant in males**

| **Chr** | **SNP** | **Pos** | **Closest Gene** | **A1** | **Males** | | | | **Females** | | | | **Males-Females Comparison** | |
| --- | --- | --- | --- | --- | --- | --- | --- | --- | --- | --- | --- | --- | --- | --- |
|  |  |  |  |  | **Freq** | **OR** | **se** | **P-value** | **Freq** | **OR** | **se** | **P-value** | **Chi-square** | **P-value** |
| **Replicated set of SNPs** | | | | | | | | | | | | | | |
| 3 | rs9882471 | 158839472 | *MFSD1* | A | 0.851 | 0.437 | 0.168 | 7.98E-07 | 0.842 | 0.864 | 0.149 | 3.24E-01 | 9.219 | **2.40E-03** |
| 5 | rs9918162 | 96987845 | *LNPEP* | T | 0.972 | 0.206 | 0.345 | 4.66E-06 | 0.966 | 2.441 | 0.516 | 8.40E-02 | 15.854 | **6.84E-05** |
| 8 | rs726411 | 130734543 | *ADCY8* | G | 0.941 | 0.344 | 0.225 | 2.16E-06 | 0.940 | 1.223 | 0.251 | 4.22E-01 | 14.155 | **1.68E-04** |
| **Non-replicated set of SNPs** | | | | | | | | | | | | | | |
| 1 | rs6413942 | 88029176 | *LOC101927891* | T | 0.163 | 2.117 | 0.164 | 4.63E-06 | 0.159 | 1.141 | 0.148 | 3.75E-01 | 7.835 | **5.12E-03** |
| 1 | rs4471236 | 88076664 | *LOC101927891* | T | 0.172 | 2.188 | 0.164 | 1.76E-06 | 0.170 | 1.037 | 0.150 | 8.09E-01 | 11.288 | **7.80E-04** |
| 1 | rs4656009 | 88080526 | *LOC101927891* | A | 0.171 | 2.141 | 0.164 | 3.58E-06 | 0.169 | 1.046 | 0.150 | 7.65E-01 | 10.385 | **1.27E-03** |
| 1 | rs10737721 | 88083118 | *LOC101927891* | T | 0.171 | 2.142 | 0.164 | 3.53E-06 | 0.169 | 1.042 | 0.150 | 7.84E-01 | 10.491 | **1.20E-03** |
| 2 | rs17754357 | 79173571 | *REG3A* | G | 0.937 | 0.361 | 0.222 | 4.49E-06 | 0.939 | 1.074 | 0.233 | 7.59E-01 | 11.512 | **6.91E-04** |
| 2 | rs116799779 | 105728304 | *NCK2* | G | 0.975 | 0.242 | 0.296 | 1.71E-06 | 0.973 | 1.371 | 0.399 | 4.28E-01 | 12.181 | **4.83E-04** |
| 2 | rs13016495 | 203581104 | *RAPH1* | C | 0.877 | 0.361 | 0.213 | 1.72E-06 | 0.865 | 0.968 | 0.192 | 8.63E-01 | 11.836 | **5.81E-04** |
| 3 | rs11127861 | 84694935 | *LINC00971* | G | 0.667 | 0.433 | 0.181 | 3.72E-06 | 0.669 | 1.234 | 0.142 | 1.38E-01 | 20.781 | **5.15E-06** |
| 3 | rs34925435 | 129947221 | *TRH* | C | 0.938 | 0.367 | 0.219 | 4.52E-06 | 0.938 | 1.259 | 0.252 | 3.60E-01 | 13.680 | **2.17E-04** |
| 3 | rs28385458 | 158837475 | *MFSD1* | G | 0.850 | 0.443 | 0.167 | 1.06E-06 | 0.842 | 0.850 | 0.148 | 2.72E-01 | 8.544 | **3.47E-03** |
| 3 | rs7616299 | 158875391 | *MFSD1* | G | 0.849 | 0.451 | 0.166 | 1.47E-06 | 0.839 | 0.798 | 0.145 | 1.20E-01 | 6.717 | **9.55E-03** |
| 3 | rs4679840 | 158876010 | *MFSD1* | A | 0.850 | 0.450 | 0.166 | 1.41E-06 | 0.839 | 0.798 | 0.145 | 1.20E-01 | 6.743 | **9.41E-03** |
| 4 | rs28384174 | 6959380 | *TBC1D14* | G | 0.941 | 0.337 | 0.238 | 4.75E-06 | 0.938 | 1.535 | 0.289 | 1.39E-01 | 16.420 | **5.07E-05** |
| 4 | rs6838042 | 6971322 | *TBC1D14* | G | 0.942 | 0.331 | 0.238 | 3.30E-06 | 0.942 | 1.409 | 0.288 | 2.34E-01 | 15.081 | **1.03E-04** |
| 4 | rs7686186 | 6984367 | *TBC1D14* | C | 0.941 | 0.336 | 0.238 | 4.67E-06 | 0.939 | 1.504 | 0.287 | 1.55E-01 | 16.112 | **5.97E-05** |
| 4 | rs6852613 | 7033910 | *LOC100129931* | G | 0.933 | 0.338 | 0.228 | 1.94E-06 | 0.929 | 1.453 | 0.263 | 1.55E-01 | 17.576 | **2.76E-05** |
| 7 | rs77104831 | 102911421 | *FBXL13* | G | 0.979 | 0.234 | 0.306 | 2.01E-06 | 0.982 | 1.088 | 0.390 | 8.28E-01 | 9.632 | **1.91E-03** |
| 8 | rs75462348 | 18533400 | *PSD3* | G | 0.976 | 0.218 | 0.302 | 4.55E-07 | 0.978 | 1.395 | 0.428 | 4.37E-01 | 12.592 | **3.87E-04** |
| 10 | rs10795162 | 4402925 | *LINC00703* | A | 0.920 | 0.383 | 0.210 | 4.60E-06 | 0.922 | 0.970 | 0.206 | 8.80E-01 | 9.998 | **1.57E-03** |
| 10 | rs7071303 | 4428648 | *LINC00703* | C | 0.911 | 0.390 | 0.199 | 2.14E-06 | 0.914 | 1.039 | 0.202 | 8.51E-01 | 11.978 | **5.38E-04** |
| 12 | rs11044882 | 19818033 | *LOC100506393* | C | 0.476 | 2.042 | 0.154 | 3.68E-06 | 0.473 | 0.985 | 0.112 | 8.94E-01 | 14.616 | **1.32E-04** |
| 12 | rs10878688 | 67888338 | *LOC101927922* | T | 0.227 | 2.493 | 0.187 | 1.05E-06 | 0.214 | 1.056 | 0.157 | 7.28E-01 | 12.348 | **4.41E-04** |
| 12 | rs741290 | 67891362 | *LOC101927922* | T | 0.155 | 2.489 | 0.195 | 3.06E-06 | 0.148 | 1.197 | 0.175 | 3.03E-01 | 7.801 | **5.22E-03** |
| 12 | rs2904502 | 67910369 | *LOC101927922* | C | 0.220 | 2.371 | 0.188 | 4.48E-06 | 0.204 | 1.125 | 0.158 | 4.56E-01 | 9.215 | **2.40E-03** |
| 12 | rs79722180 | 67914010 | *LOC101927922* | C | 0.898 | 0.316 | 0.216 | 9.83E-08 | 0.897 | 0.763 | 0.203 | 1.83E-01 | 8.866 | **2.90E-03** |
| 12 | rs2041769 | 67916704 | *LOC101927922* | G | 0.220 | 2.371 | 0.188 | 4.48E-06 | 0.204 | 1.123 | 0.158 | 4.62E-01 | 9.259 | **2.34E-03** |
| 12 | rs2930120 | 67920032 | *LOC101927922* | A | 0.220 | 2.371 | 0.188 | 4.48E-06 | 0.204 | 1.126 | 0.158 | 4.52E-01 | 9.198 | **2.42E-03** |
| 12 | rs2160243 | 67922216 | *LOC101927922* | G | 0.220 | 2.391 | 0.189 | 4.11E-06 | 0.204 | 1.126 | 0.158 | 4.54E-01 | 9.337 | **2.25E-03** |
| 12 | rs2160244 | 67922231 | *LOC101927922* | G | 0.220 | 2.391 | 0.189 | 4.11E-06 | 0.204 | 1.128 | 0.158 | 4.47E-01 | 9.293 | **2.30E-03** |
| 13 | rs4942587 | 46888665 | *HTR2A* | A | 0.764 | 0.515 | 0.143 | 3.51E-06 | 0.758 | 0.956 | 0.088 | 6.13E-01 | 13.550 | **2.32E-04** |
| 15 | rs62037093 | 100545006 | *CERS3* | G | 0.780 | 1.948 | 0.144 | 3.45E-06 | 0.775 | 1.148 | 0.090 | 1.25E-01 | 9.750 | **1.79E-03** |
| 19 | rs2302525 | 43652448 | *PLAUR* | G | 0.950 | 0.352 | 0.228 | 4.41E-06 | 0.948 | 0.759 | 0.223 | 2.17E-01 | 5.835 | **1.57E-02** |
| 22 | rs73165159 | 41950038 | *LINC00634* | C | 0.965 | 0.298 | 0.259 | 2.98E-06 | 0.965 | 0.998 | 0.312 | 9.95E-01 | 8.867 | **2.90E-03** |
| **Meta-analysis set of SNPs** | | | | | | | | | | | | | | |
| 3 | rs9862849 | 66855351 | *KBTBD8* | C | 0.900 | 0.603 | 0.059 | 2.66E-06 | 0.898 | 0.890 | 0.068 | 1.59E-01 | 18.792 | **1.46E-05** |
| 23 | rs5969117 | 87181248 | *KLHL4* | C | 0.295 | 1.490 | 0.114 | 1.42E-06 | 0.298 | 0.984 | 0.055 | 7.87E-01 | 10.802 | **1.01E-03** |

Chr: chromosome; Pos: position of SNP based on Human Genome version 38 (hg38); A1: effect allele; Freq: frequency of effect allele; OR (se): odds ratio and its standard error based on the discovery cohort for the replicated/non-replicated sets of SNPs and based on the meta-analysis results for the meta-analysis sets of SNPs

**Table S17:** **Wald chi-square test to compare odds ratios of single-nucleotide polymorphisms (SNPs) between males and females for SNPs that were specifically significant in females**

| **Chr** | **SNP** | **Pos** | **Closest Gene** | **A1** | **Males** | | | | **Females** | | | | **Males-Females Comparison** | |
| --- | --- | --- | --- | --- | --- | --- | --- | --- | --- | --- | --- | --- | --- | --- |
|  |  |  |  |  | **Freq** | **OR** | **se** | **P-value** | **Freq** | **OR** | **se** | **P-value** | **Chi-square** | **P-value** |
| **Non-replicated set of SNPs** | | | | | | | | | | | | | | |
| 1 | rs11118774 | 221612617 | *DUSP10* | G | 0.694 | 0.987 | 0.187 | 9.42E-01 | 0.680 | 2.249 | 0.171 | 2.23E-06 | 10.557 | **1.16E-03** |
| 2 | rs11678276 | 31442242 | *XDH* | G | 0.978 | 0.860 | 0.469 | 7.48E-01 | 0.978 | 0.296 | 0.263 | 3.82E-06 | 3.918 | **4.78E-02** |
| 2 | rs34779859 | 127135192 | *BIN1* | G | 0.684 | 0.962 | 0.097 | 6.89E-01 | 0.676 | 0.640 | 0.095 | 2.53E-06 | 8.982 | **2.73E-03** |
| 4 | rs57985280 | 16033919 | *PROM1* | A | 0.960 | 0.856 | 0.340 | 6.49E-01 | 0.965 | 0.370 | 0.211 | 2.38E-06 | 4.386 | **3.62E-02** |
| 4 | rs2333450 | 176306689 | *SPCS3* | T | 0.900 | 0.896 | 0.216 | 6.13E-01 | 0.893 | 0.459 | 0.159 | 9.59E-07 | 6.210 | **1.27E-02** |
| 4 | rs72708337 | 176313432 | *SPCS3* | C | 0.901 | 0.896 | 0.216 | 6.13E-01 | 0.893 | 0.458 | 0.159 | 8.67E-07 | 6.262 | **1.23E-02** |
| 5 | rs67220167 | 5141021 | *ADAMTS16* | G | 0.898 | 1.210 | 0.309 | 5.37E-01 | 0.889 | 0.462 | 0.163 | 2.18E-06 | 7.594 | **5.86E-03** |
| 5 | rs2255273 | 5147263 | *ADAMTS16* | C | 0.104 | 0.808 | 0.309 | 4.90E-01 | 0.114 | 2.111 | 0.163 | 4.58E-06 | 7.570 | **5.94E-03** |
| 5 | rs78552253 | 82045571 | *ATG10* | C | 0.924 | 0.945 | 0.274 | 8.36E-01 | 0.921 | 0.455 | 0.170 | 3.56E-06 | 5.132 | **2.35E-02** |
| 5 | rs41271129 | 82276055 | *RPS23,ATG10* | A | 0.928 | 1.201 | 0.304 | 5.46E-01 | 0.927 | 0.443 | 0.173 | 2.39E-06 | 8.155 | **4.29E-03** |
| 6 | rs62405605**^+^** | 19330431 | *LOC101928519* | A | 0.963 | 0.842 | 0.440 | 6.95E-01 | 0.961 | 0.315 | 0.239 | 1.41E-06 | 3.841 | **5.00E-02** |
| 6 | rs41500045 | 129453482 | *LAMA2* | A | 0.970 | 0.772 | 0.393 | 5.10E-01 | 0.974 | 0.306 | 0.253 | 2.96E-06 | 3.904 | **4.82E-02** |
| 6 | rs62433246 | 154259345 | *IPCEF1* | C | 0.831 | 1.097 | 0.207 | 6.57E-01 | 0.837 | 0.533 | 0.135 | 3.32E-06 | 8.502 | **3.55E-03** |
| 6 | rs1105056 | 162032707 | *PARK2* | C | 0.830 | 0.880 | 0.192 | 5.03E-01 | 0.828 | 0.544 | 0.130 | 3.12E-06 | 4.279 | **3.86E-02** |
| 7 | rs7779578 | 148973 | *LOC100507642* | C | 0.895 | 1.225 | 0.260 | 4.35E-01 | 0.898 | 0.458 | 0.156 | 5.97E-07 | 10.491 | **1.20E-03** |
| 8 | rs1062851**^+^** | 86558500 | *CPNE3* | C | 0.384 | 0.830 | 0.156 | 2.33E-01 | 0.387 | 0.565 | 0.125 | 4.94E-06 | 3.698 | **5.45E-02** |
| 8 | rs62510850**^+^** | 86562006 | *CPNE3* | C | 0.374 | 0.815 | 0.158 | 1.94E-01 | 0.380 | 0.560 | 0.126 | 4.18E-06 | 3.465 | **6.27E-02** |
| 8 | rs7000333**^+^** | 86565903 | *CPNE3* | C | 0.385 | 0.826 | 0.156 | 2.19E-01 | 0.387 | 0.565 | 0.125 | 4.87E-06 | 3.607 | **5.75E-02** |
| 8 | rs73327271 | 119128360 | *COLEC10* | A | 0.976 | 1.216 | 0.527 | 7.11E-01 | 0.975 | 0.261 | 0.247 | 5.24E-08 | 6.999 | **8.15E-03** |
| 9 | rs1359176 | 9750139 | *PTPRD* | C | 0.607 | 1.048 | 0.141 | 7.38E-01 | 0.606 | 0.595 | 0.113 | 4.11E-06 | 9.863 | **1.69E-03** |
| 9 | rs13292007 | 36977215 | *PAX5* | T | 0.782 | 1.007 | 0.179 | 9.68E-01 | 0.778 | 0.567 | 0.120 | 2.04E-06 | 7.150 | **7.50E-03** |
| 10 | rs41306812 | 13644230 | *FRMD4A* | C | 0.986 | 2.420 | 1.030 | 3.91E-01 | 0.987 | 0.195 | 0.349 | 2.78E-06 | 5.367 | **2.05E-02** |
| 10 | rs17484254 | 29259356 | *LYZL1* | T | 0.938 | 1.297 | 0.315 | 4.09E-01 | 0.941 | 0.363 | 0.195 | 1.91E-07 | 11.855 | **5.75E-04** |
| 10 | rs1538431 | 84998753 | *CCSER2* | C | 0.871 | 0.941 | 0.222 | 7.86E-01 | 0.883 | 0.515 | 0.143 | 3.43E-06 | 5.207 | **2.25E-02** |
| 11 | rs1938923 | 86640728 | *ME3* | C | 0.207 | 0.919 | 0.118 | 4.74E-01 | 0.213 | 1.637 | 0.106 | 3.41E-06 | 13.232 | **2.75E-04** |
| 12 | rs10876280 | 52363129 | *KRT85* | C | 0.569 | 0.829 | 0.149 | 2.10E-01 | 0.551 | 1.734 | 0.120 | 4.45E-06 | 14.862 | **1.16E-04** |
| 14 | rs6572843**^+^** | 52397519 | *TXNDC16* | C | 0.977 | 0.591 | 0.410 | 1.99E-01 | 0.977 | 0.300 | 0.264 | 4.96E-06 | 1.934 | **1.64E-01** |
| 15 | rs325403 | 99710810 | *MEF2A* | C | 0.355 | 1.088 | 0.155 | 5.89E-01 | 0.356 | 1.706 | 0.112 | 1.79E-06 | 5.526 | **1.87E-02** |
| 16 | rs62043827 | 77671932 | *NUDT7* | C | 0.904 | 1.150 | 0.318 | 6.61E-01 | 0.910 | 0.419 | 0.183 | 2.08E-06 | 7.558 | **5.97E-03** |
| 16 | rs10514426 | 77676721 | *NUDT7* | G | 0.905 | 1.296 | 0.329 | 4.31E-01 | 0.909 | 0.430 | 0.181 | 3.02E-06 | 8.638 | **3.29E-03** |
| 19 | rs12609754 | 20972752 | *ZNF85* | T | 0.454 | 1.103 | 0.173 | 5.71E-01 | 0.431 | 0.488 | 0.147 | 1.12E-06 | 12.861 | **3.36E-04** |
| 20 | rs6065093 | 61343615 | *CDH4* | G | 0.800 | 1.152 | 0.230 | 5.39E-01 | 0.799 | 0.510 | 0.145 | 3.44E-06 | 8.999 | **2.70E-03** |
| 20 | rs6071592 | 61344968 | *CDH4* | G | 0.865 | 1.286 | 0.285 | 3.77E-01 | 0.863 | 0.483 | 0.159 | 4.33E-06 | 9.011 | **2.68E-03** |
| **Meta-analysis set of SNPs** | | | | | | | | | | | | | | |
| 2 | rs7561207 | 69138666 | *ANTXR1* | A | 0.053 | 1.202 | 0.182 | 3.07E-01 | 0.055 | 0.470 | 0.066 | 4.08E-06 | 23.483 | **1.26E-06** |
| 4 | rs17675640 | 5095813 | *STK32B* | G | 0.657 | 1.087 | 0.070 | 2.22E-01 | 0.667 | 0.769 | 0.040 | 1.89E-06 | 18.493 | **1.71E-05** |
| 4 | rs6838792 | 5096839 | *STK32B* | C | 0.606 | 1.036 | 0.063 | 5.83E-01 | 0.617 | 0.772 | 0.040 | 1.61E-06 | 15.740 | **7.27E-05** |
| 4 | rs895681 | 5099404 | *STK32B* | T | 0.604 | 1.038 | 0.063 | 5.65E-01 | 0.617 | 0.776 | 0.040 | 2.72E-06 | 15.123 | **1.01E-04** |
| 6 | rs11969759 | 32053353 | *TNXB* | C | 0.945 | 0.909 | 0.106 | 4.72E-01 | 0.942 | 0.616 | 0.057 | 2.10E-06 | 10.410 | **1.25E-03** |
| 6 | rs10947230 | 32056618 | *TNXB* | C | 0.944 | 0.886 | 0.102 | 3.54E-01 | 0.941 | 0.619 | 0.057 | 2.33E-06 | 9.434 | **2.13E-03** |
| 6 | rs7774197 | 32078498 | *TNXB* | A | 0.943 | 0.893 | 0.103 | 3.84E-01 | 0.941 | 0.627 | 0.058 | 4.17E-06 | 9.008 | **2.69E-03** |
| 9 | rs1172922 | 90726252 | *SYK* | A | 0.116 | 1.052 | 0.096 | 6.14E-01 | 0.118 | 1.408 | 0.098 | 4.56E-06 | 4.525 | **3.34E-02** |
| 12 | rs73156187 | 131542412 | *SFSWAP* | G | 0.898 | 1.061 | 0.099 | 5.63E-01 | 0.894 | 0.695 | 0.051 | 4.47E-06 | 14.552 | **1.36E-04** |
| 12 | rs7963314 | 131573284 | *SFSWAP* | G | 0.886 | 1.061 | 0.095 | 5.52E-01 | 0.881 | 0.700 | 0.049 | 2.72E-06 | 14.976 | **1.09E-04** |
| 21 | rs12386284**^+^** | 25517756 | *MIR155HG* | T | 0.242 | 1.106 | 0.082 | 2.09E-01 | 0.249 | 1.367 | 0.087 | 4.55E-06 | 3.111 | **7.77E-02** |
| 21 | rs1783012 | 25547104 | *MIR155HG* | T | 0.754 | 0.903 | 0.067 | 2.03E-01 | 0.748 | 0.732 | 0.047 | 4.59E-06 | 6.622 | **1.01E-02** |
| 21 | rs1783013 | 25547257 | *MIR155HG* | T | 0.754 | 0.907 | 0.067 | 2.23E-01 | 0.748 | 0.733 | 0.047 | 4.65E-06 | 6.838 | **8.93E-03** |
| 21 | rs926963 | 25547744 | *MIR155HG* | T | 0.754 | 0.904 | 0.067 | 2.08E-01 | 0.748 | 0.732 | 0.047 | 4.75E-06 | 6.639 | **9.98E-03** |
| 21 | rs1893650 | 25568503 | *MIR155HG* | T | 0.244 | 1.033 | 0.071 | 6.62E-01 | 0.247 | 1.320 | 0.075 | 3.94E-06 | 5.630 | **1.77E-02** |
| 21 | rs2226326 | 25569648 | *MIR155HG* | A | 0.243 | 1.026 | 0.071 | 7.28E-01 | 0.245 | 1.319 | 0.075 | 4.38E-06 | 5.918 | **1.50E-02** |
| 21 | rs2829803 | 25575998 | *MIR155HG* | G | 0.244 | 1.035 | 0.071 | 6.41E-01 | 0.247 | 1.319 | 0.075 | 4.09E-06 | 5.515 | **1.89E-02** |
| 21 | rs2829823 | 25599076 | *MRPL39* | A | 0.242 | 1.025 | 0.071 | 7.40E-01 | 0.245 | 1.320 | 0.075 | 4.06E-06 | 6.001 | **1.43E-02** |
| 21 | rs2829832 | 25601939 | *MRPL39* | T | 0.243 | 1.026 | 0.071 | 7.26E-01 | 0.245 | 1.318 | 0.075 | 4.68E-06 | 5.867 | **1.54E-02** |

Please see the description provided below Table S16.

+ denotes that the SNP did not have significant sex-specific effects.

**Figure S1: Manhattan plot of the genome-wide association results under analysis Plan 1 (males and females)**

**
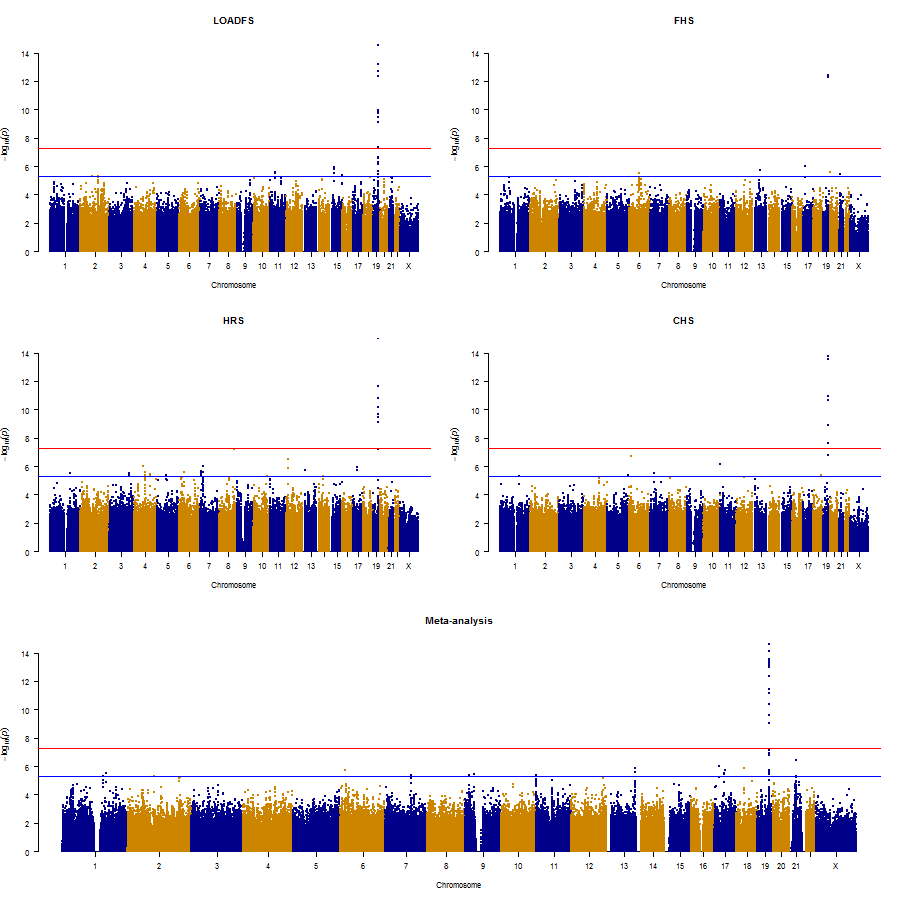
**

Red and blue lines indicate the genome-wide and suggestive significance thresholds of 5E-08 and 5E-06, respectively.

**Figure S2: QQ plot of the genome-wide association results under analysis Plan 1 (males and females)**

**
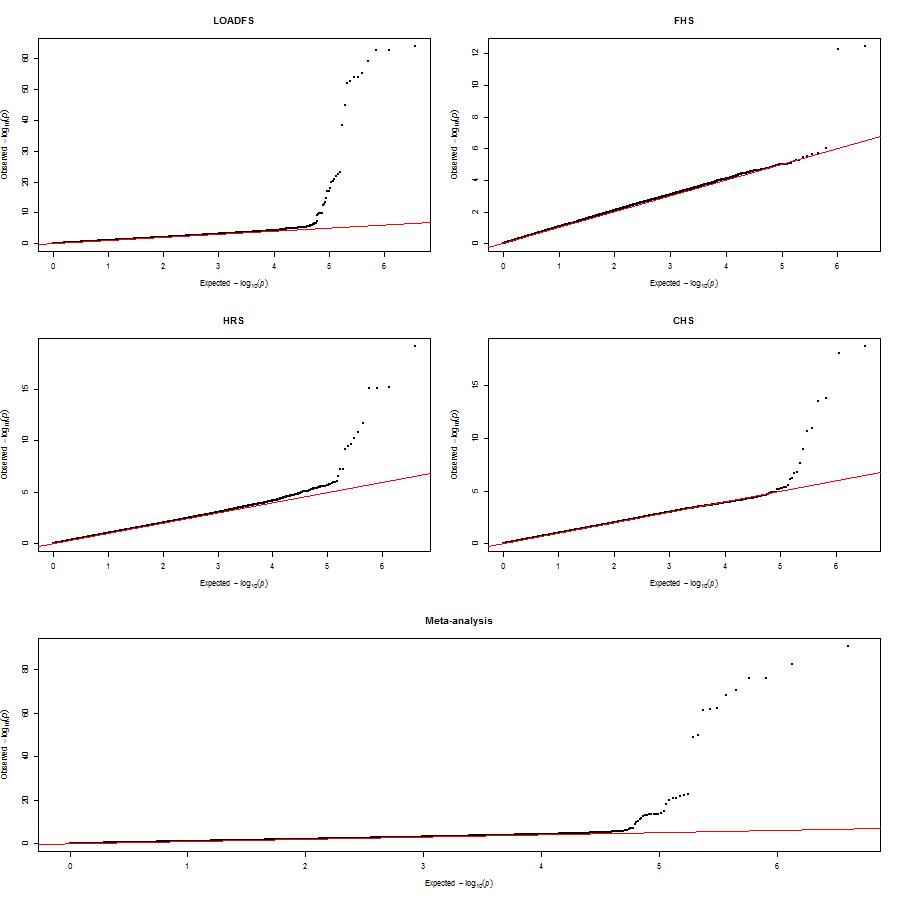
**

**Figure S3: Manhattan plot of the genome-wide association results under analysis Plan 2 (only males)**

**
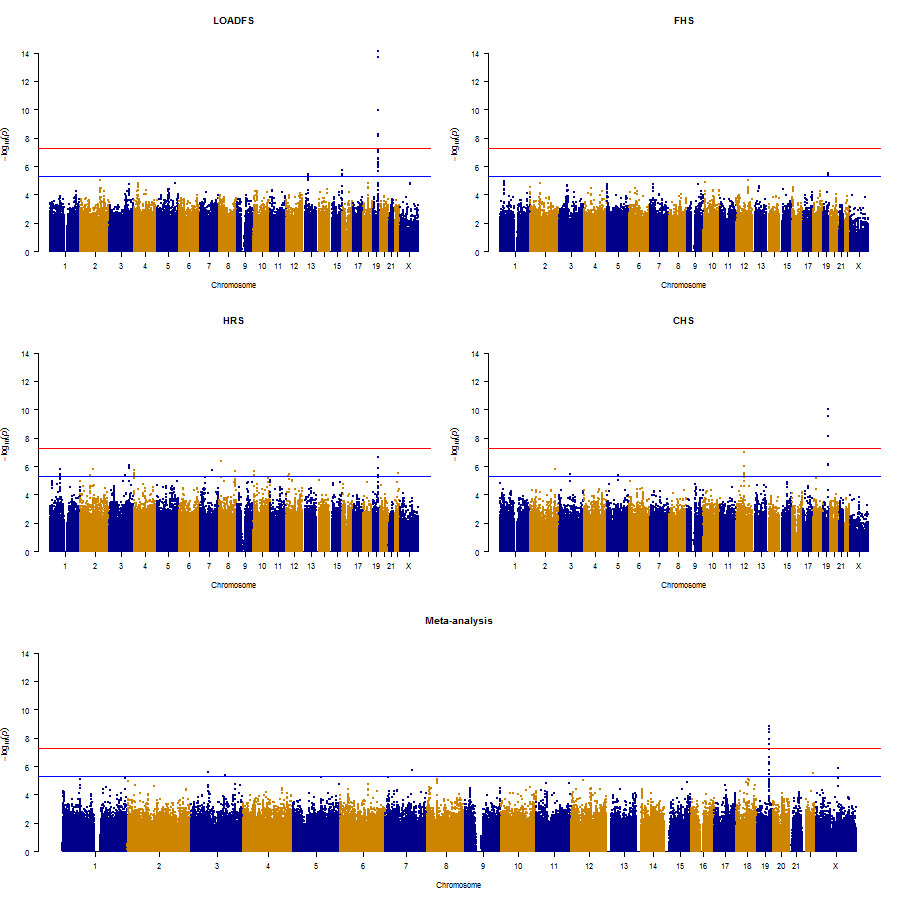
**

Red and blue lines indicate the genome-wide and suggestive significance thresholds of 5E-08 and 5E-06, respectively.

**Figure S4: QQ plot of the genome-wide association results under analysis Plan 2 (only males)**

**
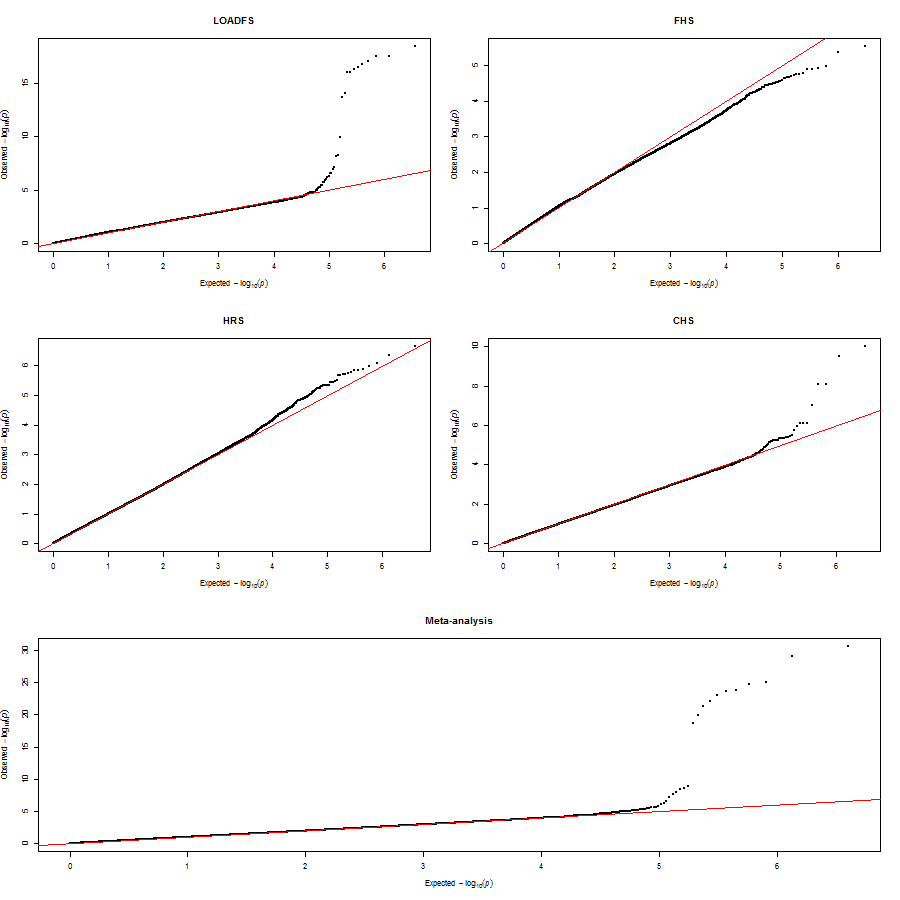
**

**Figure S5: Manhattan plot of the genome-wide association results under analysis Plan 3 (only females)**

**
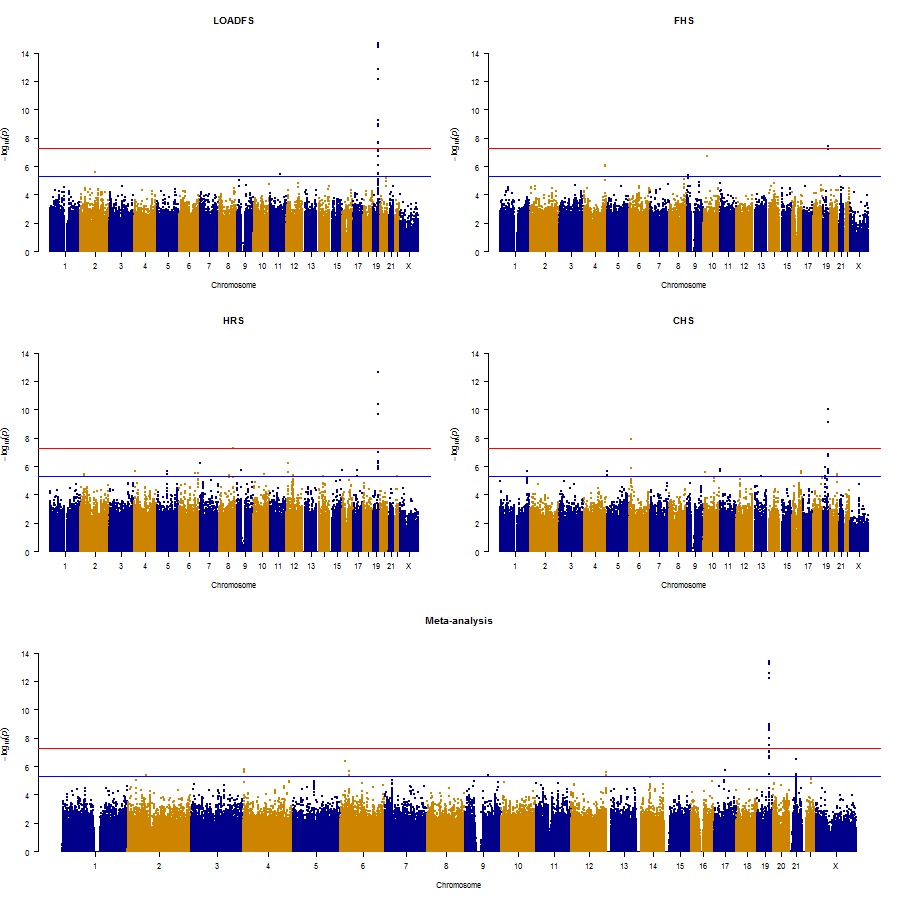
**

Red and blue lines indicate the genome-wide and suggestive significance thresholds of 5E-08 and 5E-06, respectively.

**Figure S6: QQ plot of the genome-wide association results under analysis Plan 3 (only females)**

**
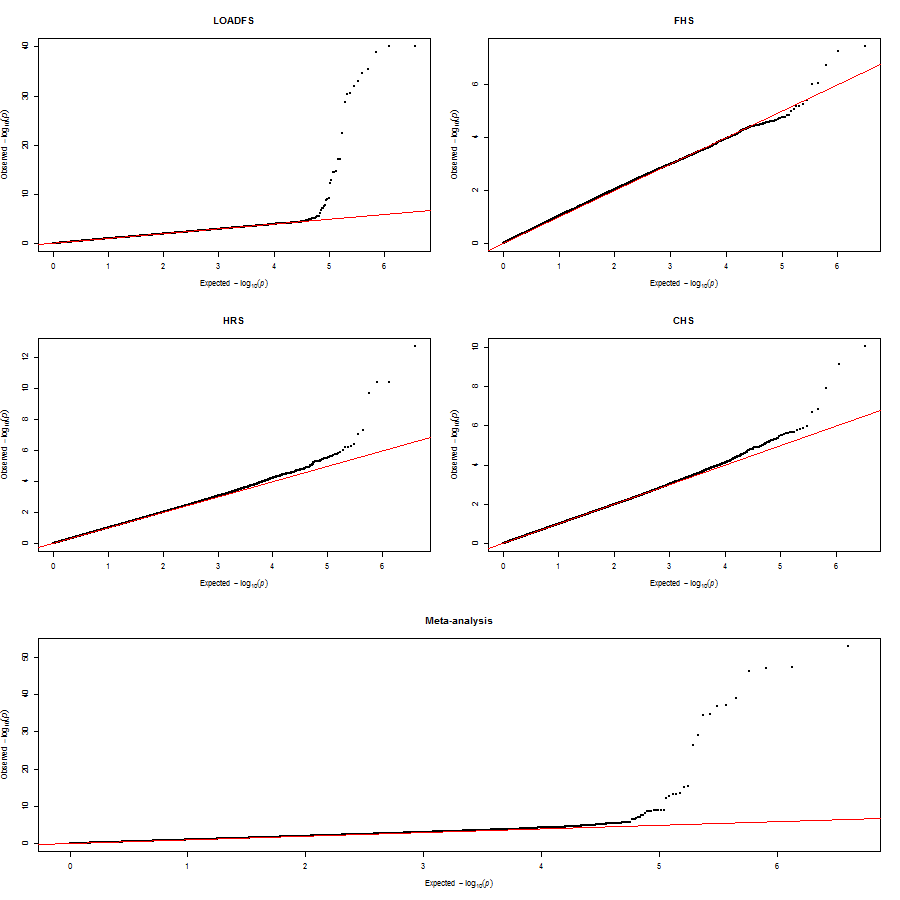
**

**Supporting Acknowledgment**

This research was supported by Grants No. P01AG043352 and R01AG047310 from the National Institute on Aging. The funders had no role in study design, data collection and analysis, decision to publish, or preparation of the manuscript. The content is solely the responsibility of the authors and does not necessarily represent the official views of the National Institutes of Health.

This manuscript was prepared using limited access datasets obtained though dbGaP (accession numbers: phs000168.v2.p2 (LOADFS), phs000007.v28.p10 (FHS), phs000287.v5.p1 (CHS), and phs000428.v2.p2 (HRS)) and the University of Michigan. Phenotypic HRS data are available publicly and through restricted access from <http://hrsonline.isr.umich.edu/index.php?p=data>.

Funding support for the Late Onset Alzheimer’s Disease Family Study (LOADFS) was provided through the Division of Neuroscience, NIA. The LOADFS includes a genome-wide association study funded as part of the Division of Neuroscience, NIA. Assistance with phenotype harmonization and genotype cleaning, as well as with general study coordination, was provided by Genetic Consortium for Late Onset Alzheimer’s Disease. This manuscript was not prepared in collaboration with LOADFS investigators and does not necessarily reflect the opinions or views of LOADFS.

The Framingham Heart Study (FHS) is conducted and supported by the National Heart, Lung, and Blood Institute (NHLBI) in collaboration with Boston University (Contract No. N01-HC-25195 and HHSN268201500001I). This manuscript was not prepared in collaboration with investigators of the FHS and does not necessarily reflect the opinions or views of the FHS, Boston University, or NHLBI. Funding for SHARe Affymetrix genotyping was provided by NHLBI Contract N02-HL-64278. SHARe Illumina genotyping was provided under an agreement between Illumina and Boston University. Funding for CARe genotyping was provided by NHLBI Contract N01-HC-65226. Funding support for the Framingham Dementia dataset was provided by NIH/NIA grant R01 AG08122. Funding support for the Framingham Inflammatory Markers was provided by NIH grants R01 HL064753, R01 HL076784 and R01 AG028321. Funding support for the Framingham C-reactive Protein dataset was provided by NIH grants R01 HL064753, R01 HL076784 and R01 AG028321. Funding support for the Framingham Adiponectin dataset was provided by NIH/NHLBI grant R01-DK-080739. Funding support for the Framingham Interleukin-6 dataset was provided by NIH grants R01 HL064753, R01 HL076784 and R01 AG028321.

The Cardiovascular Health Study (CHS) was supported by contracts HHSN268201200036C, HHSN268200800007C, N01-HC-85079, N01-HC-85080, N01-HC-85081, N01-HC-85082, N01-HC-85083, N01-HC-85084, N01-HC-85085, N01-HC-85086, N01-HC-35129, N01 HC-15103, N01 HC-55222, N01-HC-75150, N01-HC-45133, and N01-HC-85239; grant numbers U01 HL080295 and U01 HL130014 from the National Heart, Lung, and Blood Institute (NHLBI), and R01 AG-023629 from the National Institute on Aging, with additional contribution from the National Institute of Neurological Disorders and Stroke. A full list of principal CHS investigators and institutions can be found at <https://chs-nhlbi.org/pi>. This manuscript was not prepared in collaboration with CHS investigators and does not necessarily reflect the opinions or views of CHS, or the NHLBI. Support for the genotyping through the CARe Study was provided by NHLBI Contract N01-HC-65226. Additional support for infrastructure was provided by HL105756 and additional genotyping among the African-American cohort was supported in part by HL085251. DNA handling and genotyping at Cedars-Sinai Medical Center was supported in part by National Center for Research Resources grant UL1RR033176, now at the National Center for Advancing Translational Technologies CTSI grant UL1TR000124; in addition to the National Institute of Diabetes and Digestive and Kidney Diseases grant DK063491 to the Southern California Diabetes Endocrinology Research Center.

The Health and Retirement Study (HRS) genetic data is sponsored by the Genetics Resource with HRS April 21, 2010, version G Page 5 of 7 National Institute on Aging (grant numbers U01AG009740, RC2AG036495, and RC4AG039029) and was conducted by the University of Michigan. This manuscript was not prepared in collaboration with HRS investigators and does not necessarily reflect the opinions or views of HRS.

**References**

1. Lee JH, Cheng R, Graff-Radford N, Foroud T, Mayeux R. Analyses of the national institute on aging late-onset Alzheimer’s disease family study: implication of additional loci. Arch Neurol. 2008;65:1518–26.

2. Dawber TR, Meadors GF, Moore FE. Epidemiological approaches to heart disease: the Framingham study. Am J Public Health Nations Health. 1951;41:279–86.

3. Feinleib M, Kannel WB, Garrison RJ, McNamara PM, Castelli WP. The Framingham offspring study: design and preliminary data. Prev Med. 1975;4:518–25.

4. Splansky GL, Corey D, Yang Q, Atwood LD, Cupples LA, Benjamin EJ, et al. The third generation cohort of the national heart, lung, and blood institute’s Framingham heart htudy: design, recruitment, and initial examination. Am J Epidemiol. 2007;165:1328–35.

5. Fried LP, Borhani NO, Enright P, Furberg CD, Gardin JM, Kronmal RA, et al. The cardiovascular health study: design and rationale. Ann Epidemiol. 1991;1:263–76.

6. Sonnega A, Faul JD, Ofstedal MB, Langa KM, Phillips JW, Weir DR. Cohort profile: the health and retirement study (HRS). Int J Epidemiol. 2014;43:576–85.

7. Purcell S, Neale B, Todd-Brown K, Thomas L, Ferreira MAR, Bender D, et al. PLINK: a tool set for whole-genome association and population-based linkage analyses. Am J Hum Genet. 2007;81:559–75.

8. Machiela MJ, Chanock SJ. LDlink: a web-based application for exploring population-specific haplotype structure and linking correlated alleles of possible functional variants. Bioinformatics. 2015;31:3555–7.

9. Leslie R, O’Donnell CJ, Johnson AD. GRASP: analysis of genotype-phenotype results from 1390 genome-wide association studies and corresponding open access database. Bioinformatics. 2014;30:i185-194.

10. Hollingworth P, Harold D, Sims R, Gerrish A, Lambert J-C, Carrasquillo MM, et al. Common variants at ABCA7, MS4A6A/MS4A4E, EPHA1, CD33 and CD2AP are associated with Alzheimer’s disease. Nat Genet. 2011;43:429–35.

11. Lambert JC, Ibrahim-Verbaas CA, Harold D, Naj AC, Sims R, Bellenguez C, et al. Meta-analysis of 74,046 individuals identifies 11 new susceptibility loci for Alzheimer’s disease. Nat Genet. 2013;45:1452–8.

12. Naj AC, Jun G, Beecham GW, Wang L-S, Vardarajan BN, Buros J, et al. Common variants at MS4A4/MS4A6E, CD2AP, CD33 and EPHA1 are associated with late-onset Alzheimer’s disease. Nat Genet. 2011;43:436–41.

13. Miyashita A, Koike A, Jun G, Wang L-S, Takahashi S, Matsubara E, et al. SORL1 is genetically associated with late-onset Alzheimer’s disease in Japanese, Koreans and Caucasians. PLoS ONE. 2013;8:e58618.
